# A randomized crossover study of functional electrical stimulation during walking in spastic cerebral palsy: the FES on participation (FESPa) trial

**DOI:** 10.1186/s12887-021-03037-9

**Published:** 2022-01-13

**Authors:** Irene Moll, Rik G. J. Marcellis, Marcel L. P. Coenen, Sabine M. Fleuren, Paul J. B. Willems, Lucianne A. W. M. Speth, M. Adhiambo Witlox, Kenneth Meijer, R. Jeroen Vermeulen

**Affiliations:** 1 School of Mental Health and Neurosciences (MHeNs), Faculty of Health, Medicine and Life Sciences (FHML), Maastricht University, Maastricht, the Netherlands; 2 Department of Nutrition and Movement Sciences, FHML, Maastricht University, Maastricht, the Netherlands; 3 Department of Neurology, Maastricht University Medical Center (MUMC+), Postbus 5800, 6202 AZ Maastricht, the Netherlands; 4 Department of Physiotherapy, MUMC+, Maastricht, the Netherlands; 5 Adelante, Center of Expertise in Rehabilitation and Audiology, Hoensbroek, The Netherlands; 6 Department of Orthopedics, MUMC+, Maastricht, the Netherlands

**Keywords:** Cerebral palsy, Spasticity, Gait, Orthoses, Functional electrical stimulation, Goal attainment scale

## Abstract

**Background:**

Spastic cerebral palsy is the most common cause of motor disability in children. It often leads to foot drop or equinus, interfering with walking. Ankle-foot orthoses (AFOs) are commonly used in these cases. However, AFOs can be too restrictive for mildly impaired patients. Functional electrical stimulation (FES) of the ankle-dorsiflexors is an alternative treatment as it could function as a dynamic functional orthosis. Despite previous research, high level evidence on the effects of FES on activities and participation in daily life is missing. The primary aim of this study is to evaluate whether FES improves the activity and participation level in daily life according to patients, and the secondary aim is to provide evidence of the effect of FES at the level of body functions and activities. Furthermore, we aim to collect relevant information for decisions on its clinical implementation.

**Methods:**

A randomized crossover trial will be performed on 25 children with unilateral spastic cerebral palsy. Patients aged between 4 and 18 years, with Gross Motor Functioning Classification System level I or II and unilateral foot drop of central origin, currently treated with AFO or adapted shoes, will be included. All participants will undergo twelve weeks of conventional treatment (AFO/adapted shoes) and 12 weeks of FES treatment, separated by a six-week washout-phase. FES treatment consists of wearing the WalkAide® device, with surface electrodes stimulating the peroneal nerve during swing phase of gait. For the primary objective, the Goal Attainment Scale is used to test whether FES improves activities and participation in daily life. The secondary objective is to prove whether FES is effective at the level of body functions and structures, and activities, including ankle kinematics and kinetics measured during 3D-gait analysis and questionnaire-based frequency of falling. The tertiary objective is to collect relevant information for clinical implementation, including acceptability using the device log file and side effect registration, cost-effectiveness based on quality adjusted life years (QALYs) and clinical characteristics for patient selection.

**Discussion:**

We anticipate that the results of this study will allow evidence-based use of FES during walking in children with unilateral spastic cerebral palsy.

**Trial registration:**

ClinicalTrials.gov: NCT03440632.

**Supplementary Information:**

The online version contains supplementary material available at 10.1186/s12887-021-03037-9.

## Background

### Spastic cerebral palsy

Motor disability in children is most frequently caused by cerebral palsy (CP), with an incidence of 1.8 to 2.1 children per 1000 births [1, 2]. CP is defined as ‘a group of permanent disorders of the development of movement and posture causing activity limitations, resulting from an injury in the developing central nervous system’ [3–5]. In the domain of activity (based on the International Classification of Functioning, Disability and Health (ICF)), the Gross Motor Function Classification System (GMFCS) distinguishes patients with CP classified as level I (walking independently) to level V (wheelchair bound) [6, 7]. In the ICF domain of body functions and structures, the anatomical distribution (e.g. bilateral or unilateral) and the motor abnormality (i.e. spastic, dyskinetic or ataxic) are important characteristics of CP [7]. Spastic CP is the most common type of CP.

Spasticity is defined as ‘a motor disorder characterized by a velocity-dependent increase in tonic stretch reflexes (muscle tone) with exaggerated tendon jerks, resulting from hyperexcitability of the stretch reflex, as one component of the upper motoneuron syndrome’ [8, 9]. Mobility in general - and walking in particular - is often negatively affected by spasticity. Its impact can manifest in such forms as drop foot or true equinus, which can be caused by spasticity of the ankle plantar flexors (e.g. the gastrocnemius and soleus muscle), and weakness and poor selective control of the ankle dorsiflexors (e.g. the anterior tibial muscle) [10]. Consequently, patients with CP often experience fatigue, resulting in limited walking distance with subsequent a reduced physical activity level and impaired health status. Physical activity is suggested to be lower in young people with CP than in typically developing peers [11]. Patients with CP also experience an increased incidence of tripping and falling. Besides toe drag, impaired gait stability in patients with CP can cause falling [12].

### Treatment

Ankle-foot orthoses are frequently used to support the impaired ankle dorsiflexors in patients with CP and to prevent deformities [13]. However, they restrict active motions and thereby exacerbate muscle weakness of the plantar flexors [14]. Functional electrical stimulation (FES) has been named as an alternative treatment as it might function as a dynamic functional orthosis [15, 16]. The definition of FES is ‘the electrical stimulation of muscles that have impaired motor control to produce a contraction to obtain a functionally useful movement’ [17]. In our systematic review, FES has been reported to increase ankle dorsiflexion during walking (change of 2–12° at initial contact and 3–11° for peak angle in swing compared to without FES), step length and ankle dorsiflexion strength, at the level of body functions and - structures. At the level of activity and participation, there is only some evidence that FES decreases the frequency of toe-drag and improves self-perceived performance and satisfaction [15]. More evidence is needed on patient centered outcome measures. Furthermore, practical guidelines for patient selection and FES settings are needed.

### Objectives

Based on our previous systematic review [15], the overall hypothesis of the present study is that FES of the ankle dorsiflexors is an alternative to AFOs in daily life for some of the patients with CP. The primary objective is to show whether FES of the ankle dorsiflexors improves activities and participation of patients with unilateral spastic CP in daily life, measured with the Goal Attainment Scale (GAS) [18]. The secondary objective is to prove whether FES is effective at the level of body functions and structures, and activities (mainly measured with gait analysis and physical examination) and the tertiary objective is to collect relevant information on acceptability, characteristics for patient selection and cost-effectiveness to decide on implementation of FES in clinical practice. We hypothesize that the proportion of goal achievement (GAS scores) in the FES phase will be 30% higher than in the conventional phase and that FES is effective in improving ankle dorsiflexion by at least 5° during walking. Regarding characteristics for patient selection, we hypothesize that FES is effective in patients with a relatively flexible ankle joint and those who are relatively physically active, because the FES stimulation is only active during walking.

## Methods

### Study design

**Fig. 1:**
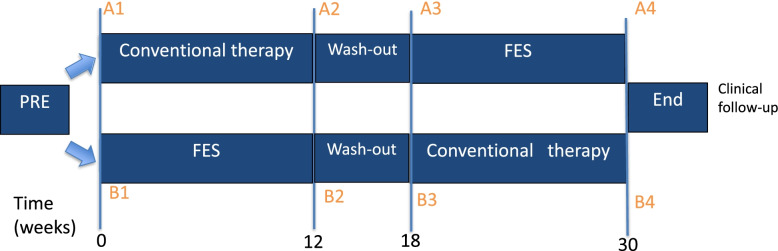
Flow chart of the two study arms, including the timing of measurements: 1) arm A starts with conventional therapy 2) arm B starts with FES. Abbreviations: FES: Functional Electrical Stimulation. Figure from the authors

The design of the study is a randomized crossover trial, consisting of a FES phase and a conventional therapy (mostly AFO) phase. It will be conducted at the Maastricht University Medical Center (MUMC+) in Maastricht, the Netherlands. The Medical Ethical Committee of the MUMC+ approved the study (METC azM/UM, study number 172033/NL63250.068.17). Figure 1 shows the flow chart for participants and the timing of the measurements. Four measurements will take place in total, at the start and end of every phase. Participants will take part in the study for 30 weeks. Patient recruitment and measurements will take place between August 2018 and August 2021.

### Participants

Subjects will be recruited from the outpatient clinics of the pediatric neurology and pediatric rehabilitation departments of the MUMC+, and collaborating centers in the region (Adelante, Center of Expertise in Rehabilitation and Audiology, Hoensbroek and Libra Rehabilitation and Audiology, Eindhoven). Individual doctors can refer patients to the MUMC+ for evaluation and participation in the study. Patients are eligible for inclusion if they are between 4 and 18 years and diagnosed with unilateral spastic CP, GMFCS level I or II (walking independently). Patients should have an unilateral foot drop of central origin with in particular the absence of initial heel contact. To avoid interference in the results of other treatments, patients are not included if they recently underwent surgery (within 1 year) or botulinum toxin injections (within 6 months). Whether the patients received injections or surgeries in the past will be reported as patient characteristic. Table 1 shows the selection criteria for participants. All participants and/or caregivers (if the participant is < 16 years) will sign an informed consent form before participating in the study. A flow chart according to the CONSORT 2010 statement will be provided showing the numbers of patient in every phase and analysis of the study [19].

**Table 1:** Overview of inclusion and exclusion criteria for patient selection

Inclusion criteria	Exclusion criteria
• Unilateral spastic cerebral palsy	• Ankle plantarflexion contracture of > 5°with the knee extended
• GMFCS level I or II	• Botulinum toxin A injections < 6 months ago
• Age 4–18 years	• Orthopaedic surgery to the legs < 1 year ago
• Unilateral foot drop of central origin	• Uncontrolled epilepsy with daily seizures
• Treated with AFO or adapted shoes	
• Ability to walk at least 15 min	
• Confirmed cerebral abnormality on MRI	

Abbreviations: GMFCS: Gross Motor Functioning Classification System; AFO: ankle foot orthosis; MRI: magnetic resonance imaging

#### Sample size calculation

The primary outcome measure will be the GAS, a 6-point scale which will be dichotomized into ‘goal achieved’ and ‘goal not achieved’. The difference is assumed to be clinically relevant if the proportion of participants achieving the goals is 30% higher for one of the treatment phases (conventional or FES). To be able to detect such a clinically relevant difference with a power of 80%, using an alpha of 5%, we need to include at least 22 participants – based on the crossover design. Previous studies reported 0–14% dropout [20, 21]. Taking possible dropout of 14% of the participants into account, 25 participants will be included. If 10 patients completed the study, an interim-analysis will be performed to check the power for the primary outcome.

### Randomization and blinding

Subjects will be randomly assigned to start with either FES or conventional treatment. The randomization by blocks of four is based on even and uneven numbers, in envelopes created by the independent expert using www.random.org/sequences. Blocks of four are applied to ensure an equal distribution of the study phases over time which is important for FES device availability: of every four patients starting the trial, two will start with FES and two will start with the conventional therapy. The envelope will be opened once the patient has been included in the study, at the end of the intake appointment.

Blinding patients to the FES treatment is not possible, as most patients feel the electrical stimulation and obvious elicited muscle contractions are necessary for proper set-up. However, blinding of an assessor is possible: the physiotherapist (SF) that performs physical examination at every time point and assesses the GAS, will be blinded for the treatment phase. Patients and parents will be informed about the blinding procedure and instructed to take off the walking aid before visiting the physiotherapist.

### Intervention

This study will compare FES of the ankle dorsiflexors during walking to conventional treatment (AFO and/or adapted shoes).

#### FES treatment

FES of the ankle dorsiflexors will be applied using the WalkAide device (Innovative Neutronics, Austin, Texas, USA, from now on: ‘FES device’) with surface electrodes. Patients will wear the device for 12 weeks in their own living environment. Patients who have a compensation for leg length difference or arch support in their AFO, will be supplied with appropriate leg length - or arch support in the form of shoe-inlay during the FES phase. If applicable, physiotherapy can be continued as in the normal situation.

The FES device stimulates the common peroneal nerve, activating the dorsiflexor muscles of the foot during the swing phase of gait. The timing of the electrical stimulation during walking is decided by a tilt sensor that measures the inclination and the acceleration of the leg. Therefore, the FES device should be worn on the leg, just below the knee as specified below.

#### Procedure FES set-up

At the start of the FES phase, there will be an individual FES set-up meeting per patient with the physiotherapist and physician (hereafter called: the therapist, ‘he’), consisting of:

1. Electrodes positioning

The patient receives an explanation regarding the procedure. Next, the therapist identifies the head of the fibula while the patient is in supine position and he marks it with a moon-shaped line inferior-posterior of the bone. He moistens this area and uses the MiniStim peripheral nerve stimulator to identify the location with the best motor-response of the tibialis anterior muscle, by moving the MiniStim along this line, while applying a bit of pressure and gradually increasing the current. In general, a dorsiflexion motor response is needed, eventually with a bit of eversion. The therapist puts his hand on the tibialis anterior muscle to feel if contraction occurs, and marks the appropriate stimulation place. This is the position for the black electrode of the FES device, over (the deep branch of) the peroneal nerve. The red electrode is placed on the upper 1/3 of the tibialis anterior muscle belly. The therapist checks the motor response with the ‘stimulation’ button of the FES device and adjusts the electrodes position if necessary. It should be kept in mind that pressure applied on the electrodes can influence the effect.

2. Fitting of the device

**Fig. 2:**
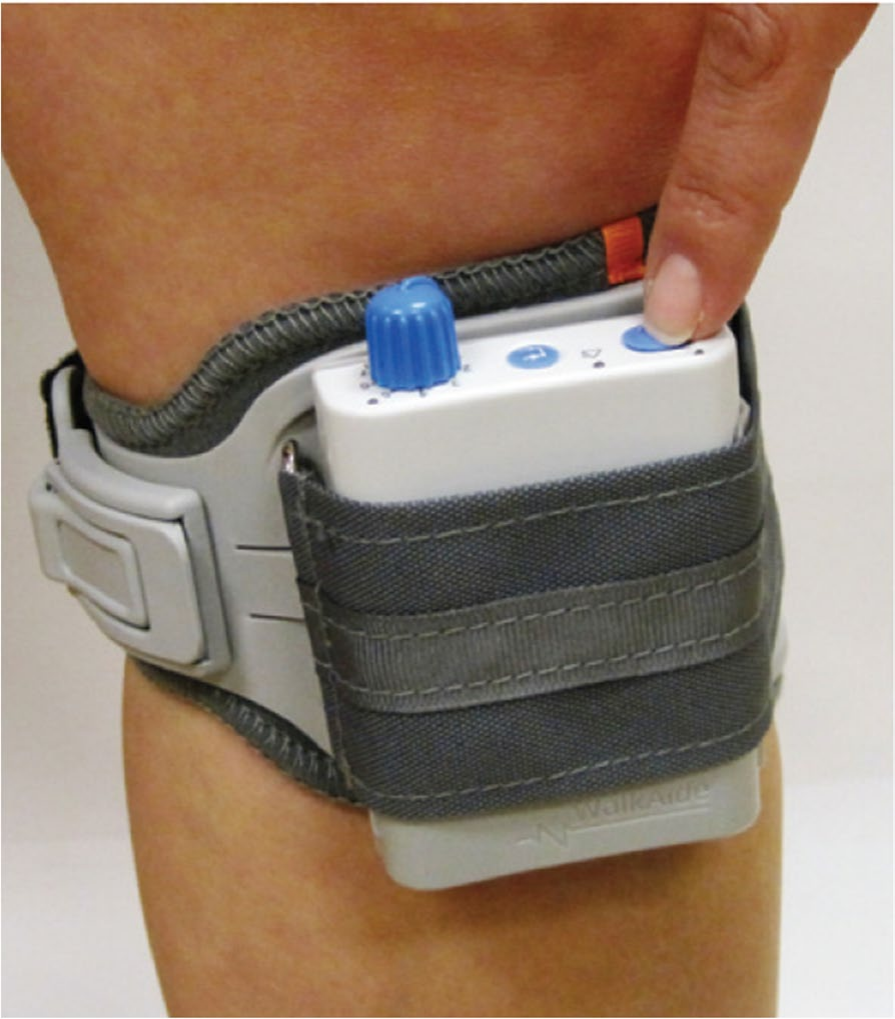
The WalkAide device positioned on the leg. The orange indicator at the top is visible. Image:© 2020 Innovative Neurotronics, Inc., All rights Reserved. Usage in paper allowed

The therapist places the cuff of the FES device (including Velcro electrode locators) over the electrodes and the device on the medial side of the lower leg. The orange indicator in the cuff (Fig. 2) should point to the middle of the patella. The position of the indicators (top and bottom of the WalkAide cuff) is marked with a surgical skin marker, to help the patient or parents put it in the correct position.

3. Timing of the stimulation

**Fig. 3:**
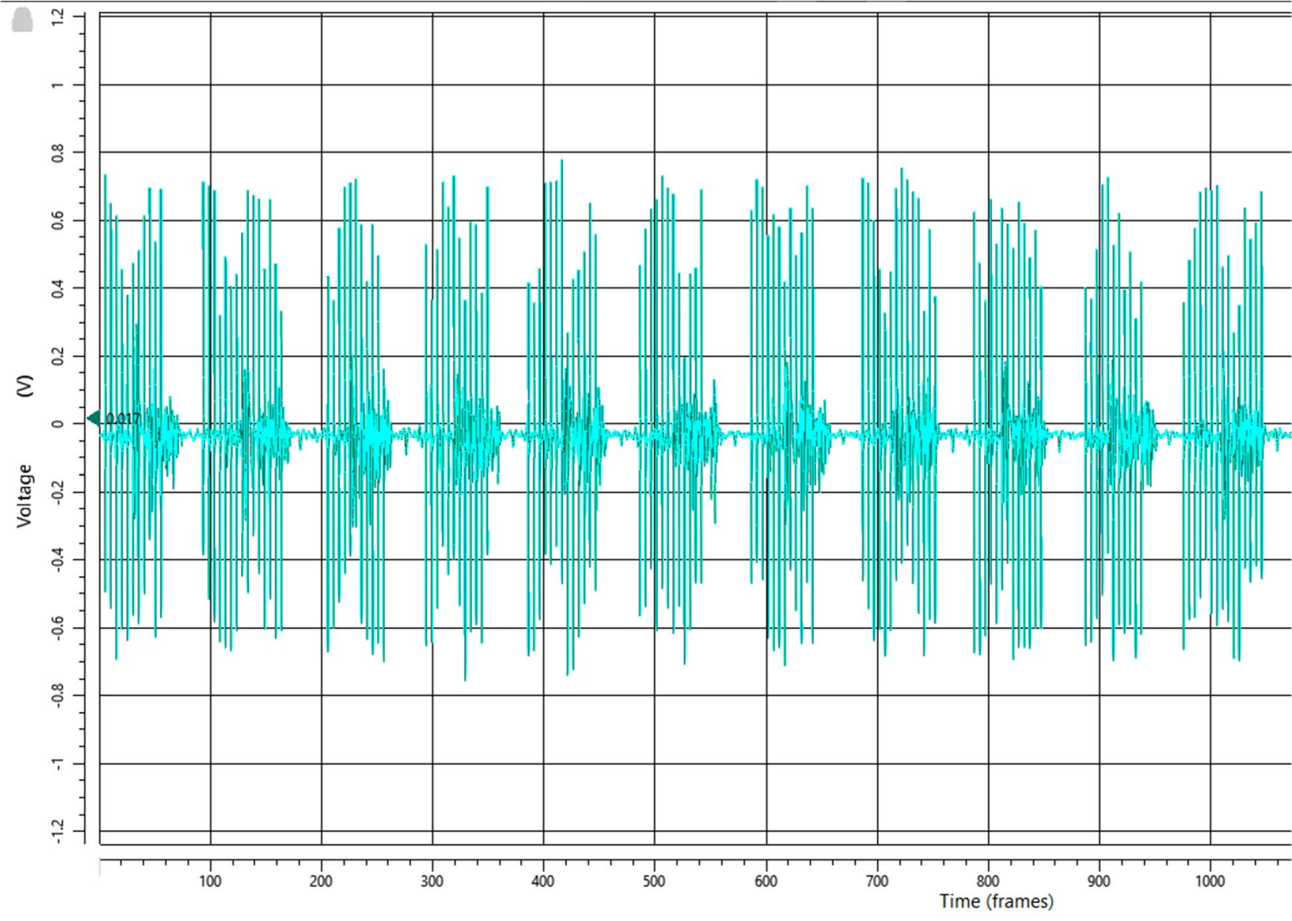
Spikes of functional electrical stimulation (FES) are visible in electromyography (EMG). X axis: 100 frames per second (100 Hz). Figure from the authors

Surface electromyography (sEMG) is used to evaluate the timing of the stimulation during the gait cycle. This is more precise than relying on the ‘auditive feedback’ from the WalkAide device that can be turned on to hear the timing of the stimulation during walking. With a quick set-up of three markers on the foot (caput of metatarsal bone 2 and 5 and the heel) and one sEMG sensor on the tibialis anterior muscle, the on/off timing of the stimulation during the gait cycle can be visualized in a 3D gait laboratory. This set-up is not used for gait analysis but only for evaluation of the timing of the stimulation. Figure 3 shows the FES stimulation in the EMG as big spikes. The goal is to let the stimulation start just after toe-off and let it stop just after heel strike. In the WalkAnalyst software, it is possible to adjust the thresholds for on/off and the durations of stimulation and no stimulation.

4. Stimulus intensity

**Fig. 4:**
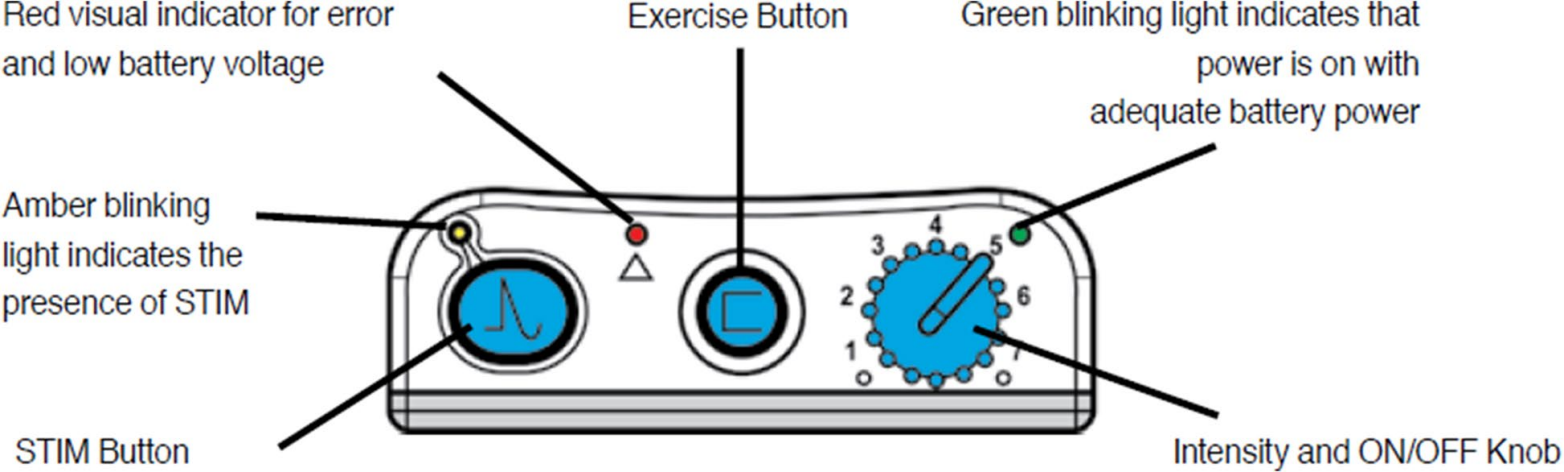
Buttons on the WalkAide device. Image:© 2020 Innovative Neurotronics, Inc., All rights Reserved. Usage in paper allowed

Patients need time to get used to the electrical stimulation. Therefore, it is advised to gradually increase the stimulus intensity and the wearing time in the first four weeks: at the start, regular breaks from the stimulation are advised every hour. This can be done by turning the device off, or sitting still, because the device won’t stimulate without movement. The use of FES can be gradually increased to 6–8 h a day. The stimulus intensity of the WalkAide device can be turned up from zero to eight (maximum 121–200 mA depending on the resistance. The resistance mainly depends on the condition of the electrodes: new, clean electrodes have lower resistance and maximum stimulus of 200 mA.) (Fig. 4). At the set-up meeting, the effective stimulus intensity, i.e. the intensity that elicits a visible muscle contraction, will be determined, both in standing and walking condition. The patient decides on the intensity that is comfortable for this moment. The therapists explains the patient to increase the intensity in the coming days to weeks if necessary. Further explanation on the device, including side effects such as skin irritation, are provided, together with the device manual and spare batteries (AA batteries).

5. Follow-up

A check-up appointment will be scheduled 2 weeks after the initial set-up. Patient and parents are asked about their positive and negative experiences. Device positioning by the patient or parents will be verified, the skin is checked for irritations and the stimulus intensity is reviewed.

#### Wash-out phase

The FES phase and the conventional treatment phase will be separated by a six-week wash-out phase, because FES therapy could have therapeutic (i.e. still present without wearing the device) effects that last for a few weeks, such as an increased muscle volume [22]. However, muscle volume is not an outcome measure in this protocol. Based on previous findings, we conclude that after six weeks without FES, no remaining effects on ankle kinematics and spatiotemporal parameters should be expected and therefore our (primary and secondary) outcome measures can reliably be measured after six weeks [22, 23]. Patients can apply their conventional treatment in the wash-out phase, since AFOs/adapted shoes are not expected to cause long-lasting therapeutic changes in patients who use them for years, and it is not ethically responsible to withhold a patient a walking-aid for six weeks.

#### Conventional treatment

The conventional treatment consists of the treatment that the patient currently receives. For most patients this will consist of:

- AFO and/or
- adapted shoes and/or
- physiotherapy

Patients not wearing an AFO for some reason, are also eligible to participate in the study.

Since this study compares FES treatment with conventional treatment, which is mostly AFO treatment, information about the AFO is needed. Therefore, type of AFO (static or dynamic) will be registered and the shank-to-vertical angle (SVA) will be calculated. The SVA is a parameter of the tuning of the AFO-footwear combination and should be calculated in midstance [24]. The line from the lateral epicondyle (knee joint center in sagittal plane) to the lateral malleolus is used as the shank for the calculation of the angle with the vertical [25]. According to the literature, the SVA should be between 7° and 15° in midstance, ideally 10°-12°, to align the ground reaction force to the joint rotation center.

### Outcome measures

**Fig. 5:**
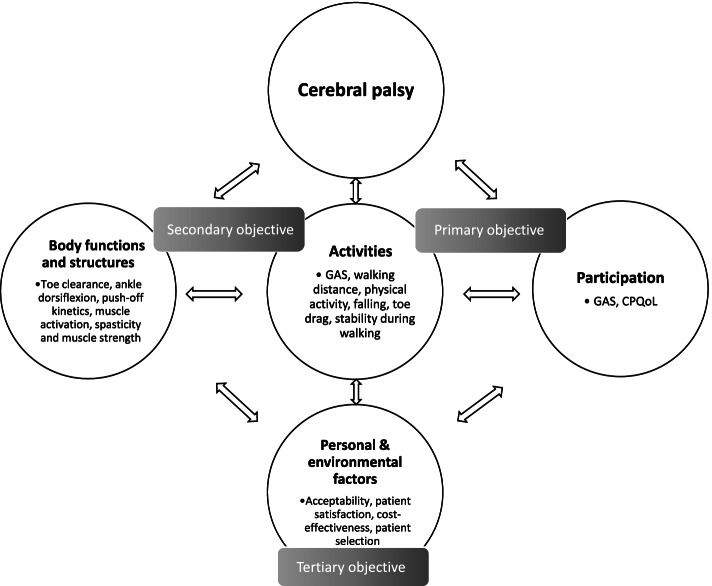
The outcome measures organized in the ICF model. Abbreviations: GAS: Goal Attainment Scale; CPQoL: Cerebral Palsy Quality of Life Questionnaire. Figure from the authors

Outcomes are grouped into the domains of the International Classification of Functioning and Health: A) Participation, B) Activities and C) Body functions & structures [7]. Fig. 5 gives an overview of the different outcome measures per domain.

### Primary objective: outcome measures in the domains of activities and participation (domains A and B)

#### Goal attainment scale (domain A and B)

The Goal Attainment Scale (GAS) is an individual outcome measure that can both describe effects per person and in groups [26]. It measures the achievement of individual, relevant goals on a 6-point scale (− 3; deterioration, till + 2; way more than the goal) [27]. The GAS is reported to be more sensitive to change than standardized functional measures [18]. Using this instrument as the main outcome measure, we want to create evidence at the level of participation and/or activities. Together with the patient, two individual goals will be prospectively defined: one aiming at the walking distance and the other one at anything that can be reflective of the FES treatment. The goals will be elaborated according to the GAS guidelines: the goal should be specific, measurable, acceptable, realistic and time-bound (SMART). The different scores (− 3 till + 2) should be clearly defined. The GAS set up and assessment will be done by a physiotherapist (SF) with previous experience regarding GAS, including training. Table 2 shows an example of the Goal Attainment Scale. The reported kappa coefficients for interrater reliability range from 0.64 to 0.89, depending on the agreements and the amount of experience with GAS [28]. For further details, we refer to previous extensive descriptions [18, 29–31]. The relevant question on this level is: *Does FES influence the achievement of individual goals in the domain of activities and participation?*

**Table 2:** Example of the goal attainment scale

Goal Attainment Scaling (GAS)	
Definition	
Setting	John is a boy of 15 years old. Because of cerebral palsy, walking is a bit more difficult for him than for most people. Especially during day trips, for example to the zoo or a theme park, he gets really tired and he is not happy with the way he walks.
Measuring method	John will score his physical performance and stamina during a day trip, on a scale from 0 to 10: 0 means ‘very bad’ and 10 means ‘perfect’.
Assignment	John, pay attention to your physical feelings and walking performance during a daytrip.
GAS levels	
−3 deterioration	Score 3: even worse: John is really sad about his stamina and walking performance and he actually can’t fulfil daytrips in a nice way, because he needs so many breaks.
−2 baseline situation	Score 4: quite bad: John is really not happy about his stamina and walking performance. He feels really tired during the day trip and needs a lot of breaks.
−1 less than the goal	Score 5: still not sufficient: John is not happy about his stamina and walking performance, but he feels a bit less tired during the day trip. He still needs regular breaks.
0 goal	Score 6: sufficient: John feels okay about his stamina and walking performance; he feels less tired during the day trip and needs less breaks.
+ 1 more than the goal	Score 7: more than sufficient: John feels a bit happy about his stamina and walking performance: he feels less tired during the day trip and needs less breaks. He can enjoy the day more.
+ 2 way more than the goal	Score 8: more than sufficient: John feels happy about his stamina and walking performance: he feels only a bit tired during the day trip. He would like to have more day trips.

#### CPQoL questionnaire - participation (domain A)

The Cerebral Palsy Quality of Life (CPQoL) Questionnaire will be used for the assessment of participation. This validated questionnaire is about an individual’s perception of their wellbeing across various domains of life. This questionnaire has good test characteristics: internal consistency (measured as Cronbach’s α) ranges from 0.74 to 0.92 for primary caregivers and from 0.80 to 0.90 for child self-report. Test-retest reliability (measured as intra-class correlation coefficient) ranges from 0.76 to 0.89 [32]. Several CPQoL versions exist: for primary caregivers of children aged 4–12 years, for children aged 9–12 year and for teenagers aged 13–18 year. The questionnaires consist of 53 to 72 questions, scored on a 9-point ordinal scale, ranging from 1; very unhappy, to 9; very happy. Dutch versions will be used. The CPQoL question ‘How do you feel about your ability to dress yourself?’ will be used for the tertiary objective. The relevant question on this level is: *Does FES influence participation as measured by the CPQoL?*

### Secondary objective – outcome measures in the domains of body functions & - structures and activities (domain B and C)

#### Walking distance (domain B)

The six-minute walk test is a common test for functional ability: it measures the distance one can walk (without running) in six minutes. Reference values have been described for spastic cerebral palsy patients and the test-retest reliability has been established [33, 34]. Although the six-minute walk test is a laboratory test, it probably reflects the walking distance in daily life. The six-minute walk test will be performed according to the guidelines [35] on a 30 m course in a hospital hallway with walking-aid. Besides the six-minute walk test, the Functional Mobility Scale (FMS) will be applied. The FMS measures the (in)dependence in walking for several distances (5, 50 and 500 m) on a 6-point ordinal scale, ranging from 1; wheelchair bound, to 6; independent on all surfaces. The interrater reliability measured as the ICC is 0.94–0.95 for the different distances [36]. The relevant questions on this level are: 1) *Does FES change the six-minute walk distance? 2) Does FES change the FMS score?*

#### Physical activity (domain B)

Physical activity is a determinant of general health. Measuring physical activity can objectively and reliably be done using acceleration-based activity monitors. The ActivPAL3 micro (PAL Technologies Ltd., Glasgow, UK) is a small activity monitor that can be attached to the upper leg in a waterproof manner, and is capable of continuous measuring for more than seven days. It has been validated in cerebral palsy patients and the agreement with video-based analysis is 97–106% [37, 38]. In this study, all participants will wear the ActivPAL for seven complete days at time point 1, 2 and 4. The device will be attached to the not-affected upper leg in a waterproof way by the researcher to stay for seven days. Seven days are measured to make sure week- and weekend days are included, as these days might show different patterns. Sitting/lying time, upright time, stepping time, and steps taken will be collected to get insight in the physical activity of the study population and to evaluate whether FES therapy makes a change in physical activity. For these data, the algorithm of the PAL software will be used. A comparable activity monitor that is developed locally, the MOX Activity Logger (MOX; Maastricht Instruments, Maastricht, The Netherlands) will be added, to provide raw acceleration data [39]. The activity monitors will be placed on the thigh of the less affected leg, vertically above each other. The relevant question on this level is: *Does FES change physical activity in daily life?*

#### Falling and toe drag (domain B)

Frequency of falling and toe drag will be collected by a 5-point ordinal scale questionnaire, validated by Pool et al. [23] Possible answers are never/sometimes/a few times a week/a few times a day/always, separately for toe drag and falling. Furthermore, a measurement for gait stability will be incorporated in the gait analysis (see below). The relevant question on this level is: *Does FES influence the frequency of falling and toe drag?*

#### Gait analysis parameters (domain B and C)

Gait analysis is performed at the ‘Computer Assisted Rehabilitation Environment’ (CAREN, MOTEK Medical, Amsterdam, Netherlands). Spatiotemporal characteristics, kinematics, kinetics, muscle activation patterns and fulfilled time of a fatigue protocol and fatigue score will be collected.

##### Equipment

The CAREN system consists of an instrumented dual-belt treadmill embedded in a mobile platform with a force plate underneath each belt and a motion-capture system based on reflective markers and 12 infrared 3D-cameras (100 Hz, 12 Bonita cameras, Vicon Nexus, Oxford, UK). A 3D virtual reality environment can be projected on a 180° cylindrical screen and three 2D video cameras are also included. The Human Body Model 2 lower limb with trunk kinematic model (HBM 2 lower limb with trunk, 26 markers, appendix A [40]) is used. Furthermore, a 16 channel surface electromyography (sEMG) system (Delsys Trigno) is used to measure muscle activity in both legs: 1. rectus femoris; 2. vastus lateralis; 3. semitendinosus; 4. biceps femoris; 5. gastrocnemius medialis; 6. soleus; 7. tibialis anterior and 8. peroneus longus. EMG sensors are placed according to the SENIAM guidelines (appendix B) [41]. All hardware is integrated in D-flow software (MOTEK Medical, Amsterdam, Netherlands).

##### Procedure

Marker placement according to the HBM2 lower limb model with trunk is done by an experienced team [40]. For the kinematic model, functional calibration of the hip as described by Camomilla [42] is applied if the patient is able to perform the movements of the ‘star arc’: swinging the leg forward, oblique sideward, sideward and to the back, and finally swinging the leg in half a circle (flexion of 30°, half circumduction to extension of 30°, neutral position). These movements are performed in the hip and the knee is extended as much as possible. Functional calibration of the knee is applied performing a flexion-extension movement five times on each side.

Gait analysis is performed with and without the walking-aid (AFO or FES). At the first measurement, the comfortable walking speed is determined (with aid), by gradually increasing the speed of the treadmill with 0.01 m/s per second, starting at 0.5 m/s. The participant is asked three times to give a sign at the comfortable speed. The mean of the results is used for all gait analyses. For five conditions, 250 steps (125 gait cycles) are measured:

1. Comfortable walking speed with walking aid
2. Fast (130% of comfortable) walking speed with walking aid
3. Comfortable walking speed without walking aid
4. Fast (130% of comfortable) walking speed without walking aid
5. Comfortable walking speed after fatigue protocol, with walking aid

Fatigue protocol: after condition 4 and a short break, the ‘fatigue protocol’ starts: the speed and incline of the treadmill gradually increase and the patient is asked to walk as long as possible wearing his/her walking aid (Table 3 shows the details). If the patient says to be really tired, the protocol is stopped: the treadmill returns to a flat position and the comfortable walking speed and the last 250 steps are measured. The patient is asked to give a score (0–10) on the children’s OMNI scale of perceived exertion for his degree of fatigue.(Appendix C, [43, 44]) The fatigue protocol is performed to investigate the influence of fatigue caused by walking on the gait pattern.

**Table 3:** Details of the fatigue protocol

Stage	Time (minutes)	% walking speed of the comfortable speed	Incline (°)	Incline (%)
1	3 (0–2:59)	70%	2	3,5
2	3 (3–5:59)	85%	4	7
3	3 (6–8:59)	100%	6	10,5
4	3 (9–11:59)	115%	8	14
5	3 (12–14:59)	135%	10	17,6
6	3 (15–17:59)	140%	12	21,3
7	3 (18–20:59)	150%	12	21,3

#### Data processing and selection

Marker and force plate data will be processed using the MOX files in custom made MATLAB software (MATLAB 9.0, R2016a, The Mathworks Inc., Natick, MA, USA). Marker tracks will be filtered with a low pass second order, zero-phase Butterworth filter, with a cut-off frequency of 12 Hz and force data will be filtered with a frequency of 20 Hz. Events (initial contact and toe off) will be calculated using combined force plate data (50 N threshold) and foot marker data (change of velocity direction) [45]. Combining these two methods will improve the accuracy of gait events detection in the center of the treadmill, triggering both force plates simultaneously. For all steps the foot marker data will be used and this timing will be corrected based on the average discrepancy between the force plate data and the foot marker data for the correct steps (triggering only one force plate). Correct kinetic steps will be selected automatically by the MATLAB software. A visual check will be performed and eventual wrong detected kinetic steps will be corrected. For EMG processing, gait events will be determined based on marker data, since force plate data is not included in the file with raw EMG signals (C3d extension). Next, all data are time-normalized into gait cycles (0–100%) based on two successive heel strikes. For each condition per participant, the approximately 125 recorded gait cycles are subsequently averaged to ensemble one average curve.

#### Spatiotemporal parameters

The following spatiotemporal parameters will be computed: walking speed (m/s), cadence (steps/min), stride time (time from initial contact to the next ipsilateral initial contact), stride length (distance between the toe marker at the second metatarsal head (MT2) and the ipsilateral MT2 marker at each initial contact in the anteroposterior direction, corrected for treadmill speed), step width (distance between the MT2 markers in mediolateral direction between both feet at initial contact) and double support time (s). Step time (s, time from one initial contact to the contralateral initial contact), stance time (s, time between initial contact and toe off of the ipsilateral leg), swing time (s, time between toe off and initial contact of the ipsilateral leg) and step length (m, distance between the toe markers in anteroposterior direction between both feet at initial contact) will be computed for right and left separately.

Minimal foot clearance (MFC) calculation is based on the second metatarsal head (MT2) marker, placed on the shoe. The height of this marker during stance phase is subtracted from the height during swing phase. This smallest relative height of MT2 during swing phase reflects the MFC. The relevant questions on this level is: *Does FES improve toe clearance?* The other spatiotemporal parameters will be used as descriptions of the gait pattern.

#### Kinematic data

Using the HBM II lower limb with trunk kinematic model with functional hip and knee calibration, kinematics in the sagittal and transversal plane of the ankle, knee, hip and trunk will be calculated based on inverse dynamics. The relevant questions on this level are: *1) Does FES improve ankle dorsiflexion in swing phase, especially in mid-swing and at initial contact? 2) Does FES improve the ankle range of motion during walking, from maximal plantarflexion pre-swing to maximal dorsiflexion during swing? 3) Does FES change ankle kinematics in the transversal plane (foot progression angle)?* Data of hip and knee will be used for general description of gait pattens and will be taken in to account for describing changes in MFC.

#### Kinetics

Force plate data and kinematic data will be synchronized. For the ankle, the moment (dorsiflexion/plantarflexion) and power (generation/absorption) will be calculated over the gait cycle. The relevant question on this level is: *Does FES change ankle plantarflexion force at push-off during gait, compared to AFO or no walking aid?*

#### Stability

Gait stability is a complex task and several measurements exist, of which the margin of stability (MoS) is one, measuring the minimum distance from the velocity-extrapolated center of mass to the boundaries of the base of support.(46)The MoS is calculated as the difference between the boundary of the base of support (BoS), based on the lateral malleoli markers, and the extrapolated center of mass [46]. The anteroposterior MoS is calculated at initial contact and the treadmill belt velocity is taken into account. For the mediolateral MoS the smallest value during a step is used. The relevant questions on this level is: *Does FES change stability during walking?*

#### EMG

The raw data (C3d file) from the gait analyses will be exported and loaded into MATLAB. The raw sEMG signals are band-pass filtered at 10 to 500 Hz using a 4th order Butterworth filter to remove low frequency motion artefacts and high frequency noise [47]. Subsequently, a linear envelope of the data is produced using full wave rectification and root mean square average with a 100 ms time window. The sEMG amplitudes are, within each gait cycle, normalized to the peak amplitude of the muscle. For each participant, the approximately 125 recorded gait cycles are subsequently averaged to ensemble one average curve of muscle activity. The onset and duration of the muscle activity are determined based on this curve. A muscle is considered to be active if the amplitude value exceeded 20% of the peak activation [47]. This protocol will be applied for each muscle. To compare several measurements, the burst duration similarity index (BDSI) will be determined [48]. This index indicates the percentage that the muscle activation patterns in two of the measurements match during the gait cycle.

In another analysis, median frequency and amplitude of the EMG per gait cycle during and after the fatigue protocol will be analysed to explore signs of fatigue.

The relevant question on this level is: *Does FES decrease muscle activation of the calf muscles?*

#### Physical examination parameters (domain C)

A permanent team of a pediatric physiotherapist and a medical doctor performs the physical examination during the assessments. The physical examination consists of the following measurements:

- Passive - and active ranges of motion of the joints of the lower limb are measured with a goniometer, and angle of muscle reaction (no resistance, resistance, catch, clonus or immobile joint) according to the Modified Tardieu Scale for spasticity [49, 50].
- Selective control assessment of the lower extremity: the patient is asked to perform the following movements, one by one, left and right separately, without moving any other part of the body: hip flexion (in supine position), knee extension (sitting), ankle dorsiflexion (both supine and sitting) and eversion (sitting) of the foot. The score ranges from 0 (unable) to 2 (normal), score 1 means ‘impaired’ [51, 52].
- At the first appointment, anthropometric and static characteristics are collected, including body length and leg length (in centimeters), weight (in kilograms), valgus/varus deformity of knee and foot (more or less than 5°), and shape of scoliosis if present.

Appendix D shows the form used for the physical examination at the first appointment.

These characteristics are potential factors that may influence the efficacy of FES treatment and therefore, they will be registered and the relation with the final main results will be analyzed. Another relevant question on this level is: *Does FES decrease spasticity of the calf muscles?*

#### Strength of plantar- and dorsiflexors (domain C)

Hand-held dynamometry with the MicroFET2 device (Hoggan scientific, Salt Lake City, USA)) is used to measure the strength of the ankle plantarflexor muscles, both with straight knee (gastrocnemius muscle) and with knee flexed in 90° (soleus muscle), and the ankle dorsiflexor muscles [53, 54]. The lever arm between the lateral malleolus and the middle of the height of the MicroFET2 is measured in meters at the lateral side of the foot. The patient is stabilized and asked to gradually build up the force (‘make method’). The mean force value in Newton of three measurements is used to calculate the moment in Newtonmeter. At least 30 s of rest between measurements of the same muscle is provided. If the values differ more than 20%, a fourth measurement is performed to replace the devious value. For hand-held dynamometry in young children with cerebral palsy, smallest detectable differences of 9–30% are reported when using average values over at least two test occasions and 2–3 repetitions per muscle [54].

The relevant question on this level is: *Does FES change the force of the dorsiflexor and plantarflexor muscles of the foot, measured by hand-held dynamometry?*

### Tertiary objective: outcome measures relevant for clinical implementation

#### Compliance, acceptability and side effects

The compliance to and acceptability of FES therapy will be derived from delivered stimulations and hours of wear time in the log file of the FES device. Side effects, such as skin lesions, discomfort or not inability to tolerate the stimulation will be registered in the database.

#### Patient satisfaction

The satisfaction of patients with their walking aid will be measured using a visual analogue scale (VAS) with 6 smileys ranging from ‘worst’ to ‘best’.

Besides this VAS scale, the feelings about donning and doffing including footwear, measured in each phase of the study (AFO versus FES), will be taken in to account. We will use the question ‘How do you feel about your ability to dress yourself?’ from the CPQoL (as explained above) for this purpose and we will explain to the participants that footwear is included in this question about dressing.

#### Cost-utility and cost-effectiveness

The EuroQol Five Dimensions Health Questionnaire Youth (EQ-5D-Y) is a child friendly and reliable instrument to measure health related quality of life [55]. Percentages of agreement in test–retest reliability range between 69.8 and 99.7% [56]. It consists of six multiple choice questions (answers: no problems/some problems/a lot of problems with…) and a scale from 0 to 100 to grade the (subjective) health of the current day. The EQ-5D-Y will be used to calculate quality adjusted life years (QALYs) to analyze cost-utility. For the cost-effectiveness analysis, the additional costs of FES therapy are compared with the additional health effects, measured by the GAS (the primary outcome measure).

#### Characteristics for patient selection

The influence of the following aforementioned parameters on the primary outcome measure will be analyzed, as these could be relevant characteristics for selection of eligible patients: age, physical characteristics (ankle range of motion in sagittal plane, foot clearance, amount of spasticity), a history of botulinum toxin injections or surgeries, physical activity in daily life (stepping time and steps taken) and 6MWT distance (in meters). Besides, the characteristics of the MRI lesion will be taken into account:

*MRI pattern brain (domain C) -* Clinically available MRI scans will be used and classified according to the scale by Fiori et al. [57]. Type of lesion will be classified according to the classification system by Himmelmann et al. [58] The research question regarding patient characteristics is: do patients for whom FES treatment is successful have certain characteristics?

## Premature termination

### Withdrawal of an individual subject

Subjects can end participation in the study at any time, with or without providing a reason. The investigator can also decide to withdraw a subject for medical reasons. Subjects withdrawn from the study will continue to get normal clinical care and follow-up. Drop-outs will be replaced if necessary, to have at least 22 patients completing the study.

### Study monitoring board

A monitoring plan will be made with the Clinical Trial Center Maastricht (CTCM) according to the local guidelines. Monitoring will include verification of informed consents, (serious) adverse events or reactions, delegation log and signature list and verification of data against source documents and medical records. This monitoring board will provide recommendations for changes to the study protocol if necessary.

## Statistics

For all analyses, we will use IBM SPSS statistics version 24 or higher.

### Descriptive statistics

Patient characteristics including gender, age, GMFCS level, affected side, MRI pattern and ankle range of motion, and data from the gait analysis (based on 250 steps) will be described. The mean and standard deviation or median and first and third quartile will be used, depending on the distribution. The distributions will be checked using histograms and Q-Q plots.

### Primary outcome

GAS scores will be dichotomized in to ‘goal achieved’ (scores 0 to + 2) and ‘goal not achieved’ (scores − 1 to − 3). The McNemar test (non-parametric) will be applied to test whether the proportions of goal achievement are significantly different between the FES and conventional phase. Raw GAS scores will also be provided. Beside an analysis including the patients who completed the study, an intention to treat analysis will be performed including the patients who discontinued the study as ‘goal not achieved’ (‘worst case analysis’). A *p*-value of 0.05 is considered to be statistically significant in this analysis.

### Secondary outcomes

To assess the effect of FES and AFO treatment on gait over time, linear mixed-effects models will be performed, with gait analysis parameters as dependent variable and ‘walking aid’ (AFO, without AFO, FES, without FES) and ‘time’ [1 or 2] as fixed factors, and an interaction term ‘walking aid * time’ if applicable and a random intercept for subjects. A p-value of 0.05 is considered to be statistically significant in this analysis and Bonferroni correction for multiple comparisons is applied. Boxplot graphs will be made, showing the mean and standard deviation, as well as the individual data. This concerns the parameters: ankle dorsiflexion (in mid-swing and at initial contact), foot progression angle, foot clearance and push-off kinetics.

Nonparametric testing is expected to be appropriate for data with a source other than gait analysis, because of the lower number of observations (one measurement per time, instead of a mean of multiple measurements). This applies to the following parameters: dynamometry force, ankle range of motions based on physical examination, questionnaires (frequency of falling and toe drag, CPQoL, EQ5D-Y), physical activity and 6MWT distance. The Wilcoxon matched-pairs (signed ranks) test will be applied on the change-scores.

#### Characteristics for patient selection

We will identify patients in whom FES treatment was successful, based on the GAS. Next, we will review their age, MRI pattern, ankle angle (both during gait and physical examination), amount of spasticity, foot clearance, physical activity and 6MWT distance. Explorative logistic regression will be performed to see whether we can identify factors that are important for the chance of success of FES treatment.

#### Cost-effectiveness and cost-utility analysis

The McNemar test will be performed for the GAS outcome (goal achieved versus goal not achieved) for the cost-effectiveness analysis, just as in the main analysis. The Wilcoxon signed rank test will be performed for the EQ5D results for the cost-utility analysis. The time-horizon of both analyses is 3 months (i.e. referring to period before crossover). With a time-horizon of 3 months, no discounting is needed. Bootstrapping and sensitivity analyses will be performed to reflect respectively stochastic and deterministic uncertainty in the trial-based cost-effectiveness analysis. Cost-effectiveness acceptability curves will be constructed for a range of threshold values based on bootstrap analyses. Sensitivity analyses will be performed for price estimates and the perspective of the analyses. Quadrant graphs will be made showing the cost difference and effect difference, both for QALYs and GAS, including raw scores of the GAS and a line at the cut-off value for goal achieved/goal not achieved.

## Discussion

We anticipate that the results of this study will allow evidence-based use of FES during walking in children with unilateral spastic cerebral palsy. More specifically, we expect for the primary objective that FES can help part of the patients to achieve their individual goals, such as being more satisfied with their quality of walking, in the ICF domains of activities and participation. This would result in higher GAS scores for the FES phase than for the AFO phase. We expect that improvement in these domains, would be accompanied by improvements in the domain of body functions and structures (domain C, secondary objective). We expect FES to improve toe clearance, ankle dorsiflexion and push-off kinetics during walking in successful cases, without impairing plantarflexion during push-off. Finally, based on our review, we expect the acceptability of FES therapy to be good and to be preferred over AFO in some cases.

By providing clear guidelines for successful FES therapy in patients with unilateral spastic CP, we hope to make insurance coverage of FES therapy possible for suitable patients.

A limitation of this study is that blinding of the patients to the treatment is not possible, because patients can feel the electrical stimulation. However, when possible, the assessor will be blinded for the current treatment allocation. This is the case for the physical therapist performing the physical examination and assessing the GAS. Furthermore, several types of bias should be taken into account when describing limitations [59]. We expect that the study population is a representative sample of the population with unilateral spastic CP, GMFCS I-II. However, patients being not satisfied with an AFO might be overrepresented, because patients who are satisfied with an AFO could be less motivated to take part in a (time-consuming) study. This could implicate a minor selection bias. Regarding classification bias, we have minimalized the risk of classification (measurement) bias by using standardized measurements in all study phases and by implementing blinding whenever possible. Last, age could induce a confounding bias, because the acceptable FES stimulus might be lower in young patients: however, this can be fully discussed after obtaining the results.

## Dissemination policy

All patients will receive their individual results both written and oral after they completed the measurements (or quitted the study). The overall results of the trial will be published after completion of the study and patients and concerned physicians will be notified.

## Supplementary Information

**Additional file 1.**

**Additional file 2.**

## Data Availability

Data will be anonymized as much as possible (not possible for videos in the gait analysis) and stored at the study site with limited access. The datasets analysed during the current study are available from the corresponding author on reasonable request.

## Abbreviations

AFO: Ankle-foot orthosis
AFOs: Ankle-foot orthoses
BoS: base of support
CAREN: Computer Assisted Rehabilitation Environment
CP: cerebral palsy
CPQoL: Cerebral Palsy Quality of Life Questionnaire
CTCM: Clinical Trial Center Maastricht
EMG: electromyography
EQ-5D-Y: EuroQol Five Dimensions Health Questionnaire Youth
FES: functional electrical stimulation
FHML: Faculty of Health, Medicine and Life Sciences
FMS: Functional Mobility Scale
GAS: Goal Attainment Scale
GMFCS: Gross Motor Functioning Classification System
HBM2: Human Body Model 2
Hz: Hertz
ICF: International Classification of Functioning, Disability and Health
m: meter
METC: Medical Ethical Committee
MFC: minimal foot clearance
MHeNs: School of Mental Health and Neurosciences
min: minute
MoS: margin of stability
MRI: magnetic resonance imaging
MT2: second metatarsal head
MUMC+: Maastricht University Medical Center
N: Newton
QALYs: quality adjusted life years
s: second
sEMG: Surface electromyography
SENIAM: surface EMG for non-invasive assessment of muscles
SMART: specific, measurable, acceptable, realistic and time-bound
SVA: shank-to-vertical angle
USA: United States of America
VAS: visual analogue scale
3D: three dimensional
