# Supplementary material for: A randomized crossover study of functional electrical stimulation during walking in spastic cerebral palsy: the FES on participation (FESPa) trial
Source: BMC Pediatr. 2022 Jan 13;22:37. doi: 10.1186/s12887-021-03037-9 (PMC8756646; doi:10.1186/s12887-021-03037-9)
Supplement: Supplementary file 1 — Additional file 1. [file 12887_2021_3037_MOESM1_ESM.docx]

**Appendix A: Human Body Model 2 – lower limb** (37)


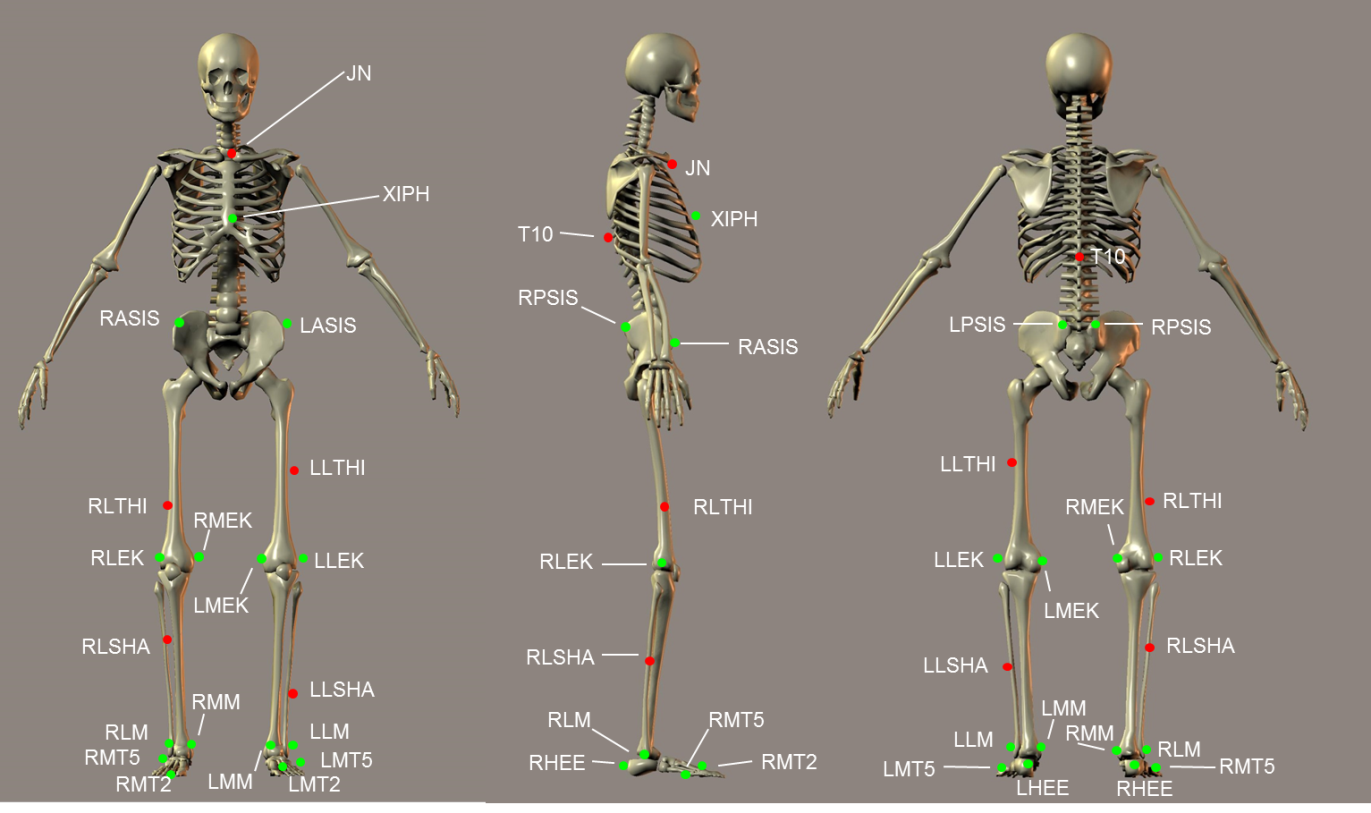
**Figure 1**: marker positions on the body

**Table 1**: marker placement explained.

| No. | Marker | Position | Placement remarks |
| --- | --- | --- | --- |
| 1 | T10 | T10 | On the 10^th^ thoracic vertebra |
| 2 | XIPH | Xiphoid process | Xiphoid process of the sternum |
| 3 | JN | Jugular notch | On the jugular notch of the sternum |
| 4 | LASIS | Pelvic bone left front | Left anterior superior iliac spine |
| 5 | RASIS | Pelvic bone right front | Right anterior superior iliac spine |
| 6 | LPSIS | Pelvic bone left back | Right posterior superior iliac spine |
| 7 | RPSIS | Pelvic bone right back | Right posterior superior iliac spine |
| 8 | LLTHI | Left thigh, lateral | 1/3 on the line between the left greater trochanter and LLEK |
| 9 | LLEK | Left lateral epicondyle of the knee | On the lateral side of the joint axis |
| 10 | LMEK* | Left medial epicondyle of the knee | On the medial side of the joint axis. Check by holding both points and bending the knee; markers should not/hardly move. |
| 11 | LLSHA | Left shank, lateral | 2/3 on the line between LLEK and LLM |
| 12 | LLM | Left lateral malleolus of the ankle | The center of the left lateral malleolus |
| 13 | LMM* | Left medial malleolus of the ankle | Most pronounced part of the left medial malleolus |
| 14 | LHEE | Left heel | Center of the heel at the same height as LMT2 |
| 15 | LMT2 | Left 2^nd^ meta tarsal | Caput of the 2^nd^ meta tarsal bone, on joint line midfoot/toes |
| 16 | LMT5 | Left 5^th^ meta tarsal | Caput of the 5^th^ meta tarsal bone, on joint line midfoot/toes |
| 17 | RLTHI | Right thigh, lateral | 2/3 on the line between the right greater trochanter and RLEK |
| 18 | RLEK | Right lateral epicondyle of the knee | On the lateral side of the joint axis |
| 19 | RMEK* | Right medial epicondyle of the knee | On the medial side of the joint axis. Check by holding both points and bending the knee; markers should not/hardly move. |
| 20 | RLSHA | Right shank, lateral | 1/3 on the line between RLEK and RLM |
| 21 | RLM | Right lateral malleolus of the ankle | The center of the right lateral malleolus |
| 22 | RMM* | Right medial malleolus of the ankle | Most pronounced part of the right medial malleolus |
| 23 | RHEE | Right heel | Center of the heel at the same height as RMT2 |
| 24 | RMT2 | Right 2^nd^ meta tarsal | Caput of the 2^nd^ meta tarsal bone, on joint line midfoot/toes |
| 25 | RMT5 | Right 5^th^ meta tarsal | Caput of the 5^th^ meta tarsal bone, on joint line midfoot/toes |

* These markers can be removed after calibration of the model.

**Appendix B: SENIAM guidelines for EMG sensor placement**(38)

| **Numbers left & right** | **Picture** | **Muscle** | **Patient position** | **Placement** | **Movement to visualize muscle** | **Direction of sensor** |
| --- | --- | --- | --- | --- | --- | --- |
| 1-  9 | 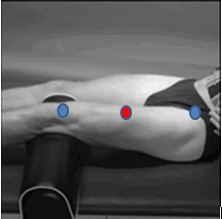 | Rectus Femoris (quadriceps) | supine | At 1/2 on the line from the anterior spina iliaca superior to the superior part of the patella | Extension of the knee | Straight |
| 2-10 | 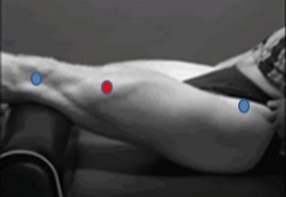 | Vastus Lateralis (quadriceps) | supine | At 2/3 on the line from the anterior spina iliaca superior to the lateral side of the patella | Extension of the knee | Oblique |
| 3-11 | 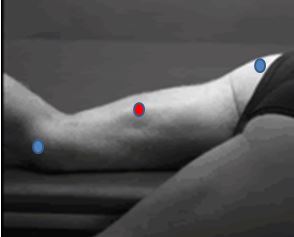 | Medial hamstrings:  Semitendinosus | prone | At 50% on the line between the ischial tuberosity and the medial epicondyle of the tibia | Flexion of the knee against pressure | Oblique |
| 4-12 | 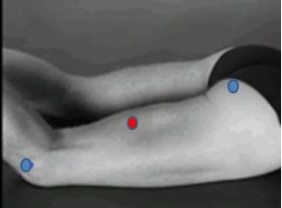 | Lateral hamstrings:  Biceps femoris | prone | At 50% on the line between the ischial tuberosity and the lateral epicondyle of the tibia | Flexion of the knee against pressure | Straight |
| 5-13 | 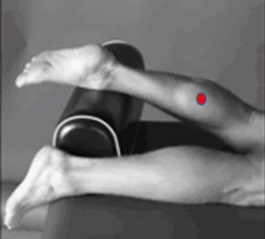 | Gastrocnemius medialis | prone | At the most prominent bulge of the muscle | Plantarflexion of the ankle with the knee extended. | Straight |
| 6-14 | 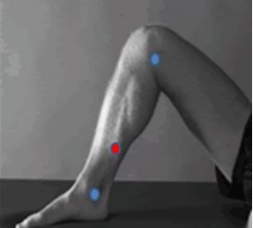 | Soleus | prone | At 2/3 of the line between the medial condylis of the femur to the medial malleolus. | Plantarflexion of the ankle with the knee flexed. | Oblique |
| 7-15 | 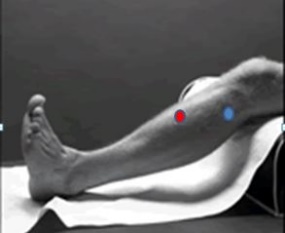 | Tibialis anterior | supine | At 1/3 on the line between the tip of the fibula and the tip of the medial malleolus. | Dorsiflexion in the ankle | Straight |
| 8-16 | 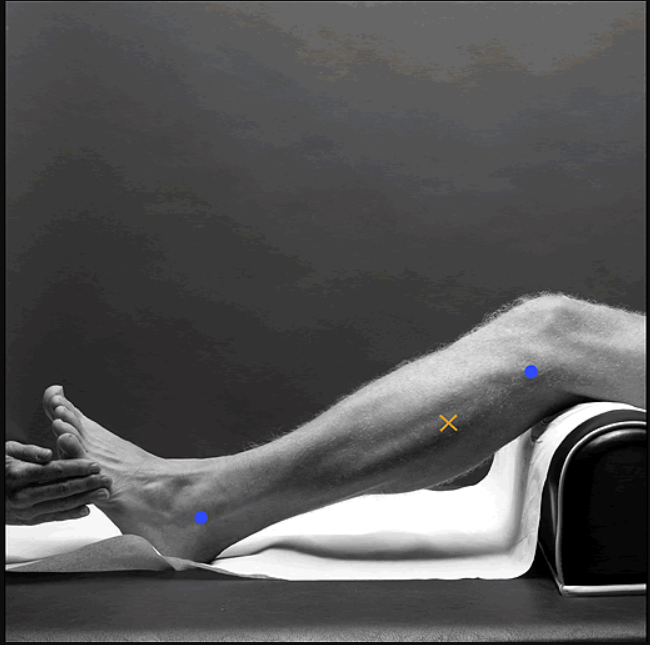 | Peroneus longus | prone | At 1/4 on the line between the tip of the head of the fibula to the tip of the lateral malleolus | Eversion and plantarflexion of the ankle | Straight |

**Appendix C: OMNI fatigue scale** (translation in Dutch: courtesy of E. Bolster)(40, 41)


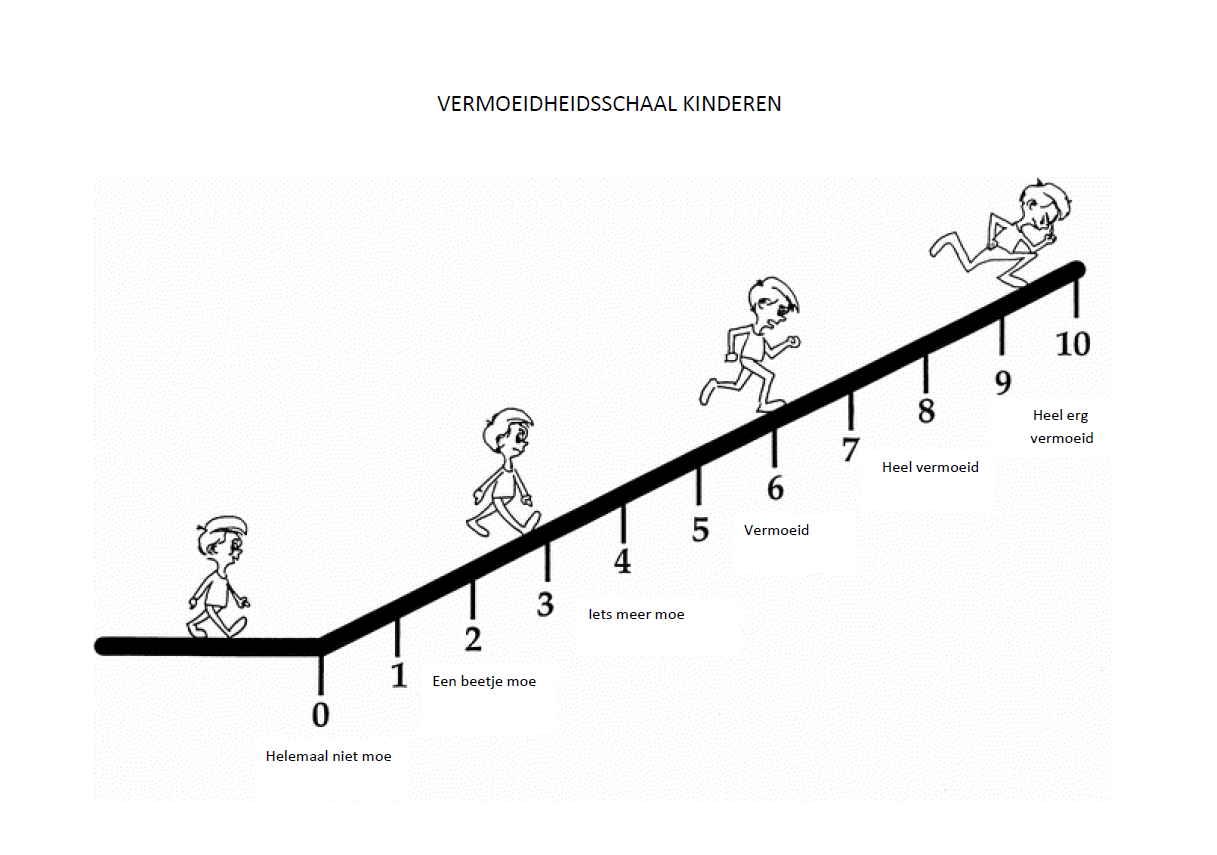


**Appendix D: Form for the first physical examination**

|  |  | Left | | | MTS quality* | Right | | | MTS quality* |
| --- | --- | --- | --- | --- | --- | --- | --- | --- | --- |
| **Movement direction** | **Position** | **1** | **2** | **3** |  | **1** | **2** | **3** |  |
| **1 = AROM (active range of motion), 2 = AOC (angle of catch), 3 = PROM-max (passive range of motion maximal).  * MTS quality = quality of muscle reaction according to the Modified Tardieu scale (0= no resistance, 1 slight resistance, 2 = catch, 3 fatigable clonus, 4 = infatigable clonus, 5 = joint immobile).** | | | | | | | | |  |
|  | **Supine position** |  |  |  |  |  |  |  |  |
| Hip extension (Thomas test) | Contralateral hip in flexion, pelvis in neutral position |  |  |  |  |  |  |  |  |
| Hip abduction 1 | Hip and knees extended |  |  |  |  |  |  |  |  |
| Hip abduction 2 | Hips extended, knees flexed, lower legs hanging off the couch |  |  |  |  |  |  |  |  |
| Knee extension (90°) (popliteal angle) | Hips and knees in 90° flexion |  |  |  |  |  |  |  |  |
| Knee extension (45°) (popliteal angle) | Hips 45° flexion and knees in 90° flexion |  |  |  |  |  |  |  |  |
| Knee extension (0°) (popliteal angle) | Hips 0° flexion and knee 90° flexion (lower legs hanging off the couch ) |  |  |  |  |  |  |  |  |
| Ankle dorsiflexion | Hips and knees in 90° flexion |  |  |  |  |  |  |  |  |
| Ankle dorsiflexion | Hip and knees extended |  |  |  |  |  |  |  |  |
|  | **Prone position** |  |  |  |  |  |  |  |  |
| Hip extension (Staheli test) | Hips 20-30° flexion |  |  |  |  |  |  |  |  |
| Hip endorotation | Prone position, legs resting at the couch |  |  |  |  |  |  |  |  |
| Hip exorotation | Prone position, legs resting at the couch |  |  |  |  |  |  |  |  |
| Knee flexion | Prone position, legs resting at the couch |  |  |  |  |  |  |  |  |

| **Statics** | | | | | | |
| --- | --- | --- | --- | --- | --- | --- |
|  |  |  | **Left** | | **Right** | |
| **Statics** | **Normal value** | **Execution** |  | |  | |
| Knee flexion |  | How much is the knee flexed (in degrees) during stance? Measure the angle femur – tibia. |  | |  | |
| Calcaneus valgus | 0-5° | Is the calcaneus in a valgus position of more than 5°? | yes | no | yes | no |
| Calcaneus varus | 0-5° | Is the calcaneus in a varus position of more than 5°? | yes | no | yes | no |
| Pes plano valgus | N/A | Does the foot show total valgus tilt with luxation of the navicular os? | yes | no | yes | no |
| Foot Rocker bottom | N/A | Does the foot show rocker bottom shape? (Congenital vertical talus: a prominent calcaneus/heel and a convexly rounded sole) | yes | no | yes | no |
| Foot antepes abductus | N/A | Is the front part of the foot in abduction compared to the hind part of the foot? | yes | no | yes | no |
| Foot antepes adductus | N/A | Is the front part of the foot in adduction compared to the hind part of the foot? | yes | no | yes | no |
| Scoliosis 1 | N/A | Select the shape of the scoliosis if applicable. | 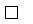 | 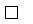 | 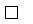 | 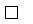 |
| Disequilibration | N/A | Is the scoliosis balanced, i.e. is C7 straight above the sacrum? Left = disequilibration to the left, right = disequilibration to the right. | yes | no | yes | no |
| Scoliosis 2 | N/A | Select the shape of the scoliosis if applicable. | 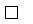 | 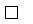 | 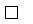 | 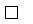 |
| Posture lumbar spine |  | Can the lordosis of the lower spine be corrected to a flat surface by bending the legs? | yes | no |  |  |
| Knee varus | 0-5° | Does the knee show varus position of more than 5° when extended? | yes | no | yes | no |
| Knee valgus | 0-5° | Does the knee show valgus position of more than 5° when extended? | yes | no | yes | no |
| Knee patella alta | 0 cm | Measure the difference between the lower border of the patella and the joint space in centimeters | cm | no | cm | no |
| Thigh foot angle | 0-20° | Hip 0°, knee 90° flexion: measure the angle between the axis of the upper leg and the line between digitus 2-3. + = exo, - = endo |  |  |  |  |
| Bimalleolar angle | 20-50° | Hip 0°, knee 0° flexion: measure the angle between the axes of both malleoli. + = exo, - = endo |  | |  | |
| Hip anteversion angle | (age dependent) | Hip 0°, knee 90° flexion: rotate till the neck of the femur is horizontal. Measure the angle between the lower leg and the vertical. |  | |  | |
| Leg length | N/A | Make sure the patient lies in a straight position. Measure the distance between the SIAS and the medial malleolus. | cm | | cm | |
| Length (cm) | N/A | Use the ruler. |  | | | |
| Weight (kg) | N/A | Use the scale. |  | | | |

| **Selectivity**  2 = good selectivity, 1 = partial selectivity, 0 = no selectivity | | | **Left** | **Right** |
| --- | --- | --- | --- | --- |
| Hip flexion | Supine | Active bending of the hip (knee extended) |  |  |
| Knee extension | Sitting | Active knee extension |  |  |
| Dorsiflexion | Supine | Active dorsiflexion of the ankle while supine |  |  |
| Dorsiflexion | Sitting | Active dorsiflexion of the ankle with hanging legs |  |  |
| Active eversion | Sitting | Active eversion of the ankle with hanging legs |  |  |

| Dynamometry | | | | | | | | | | |
| --- | --- | --- | --- | --- | --- | --- | --- | --- | --- | --- |
| **Explanation** | Ankle plantarflexor: **gastrocnemius muscle** | | | Ankle plantarflexor:  **soleus muscle** | | | | | Ankle **dorsiflexors** | |
| **Position of the patient** | Partly sitting posture, hips 45° flexed, knees 10° flexed, ankle 0° (= 90° angle with the lower leg), feet over the border of the couch. | | | Supine, hip and knees 90° flexed, the lower legs resting at the pillow block, ankle 0° (= 90° angle with the lower leg), feet over the border of the cushion. | | | | | Supine, hip and knees extended, ankle 0° (90° (= 90° angle with the lower leg) and neutral position in the frontal plane, feet over the border of the couch. | |
| **Position of the tester** | Sitting at the stool at the side of the feet of the patient, back against the wall, face towards the patient.  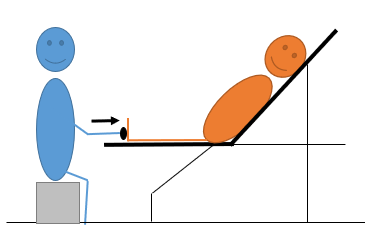 | | | Standing at the side of the feet of the patient, facing the patient, legs in stride position. Both arm fully extended, from shoulders to MicroFET in one horizontal line.  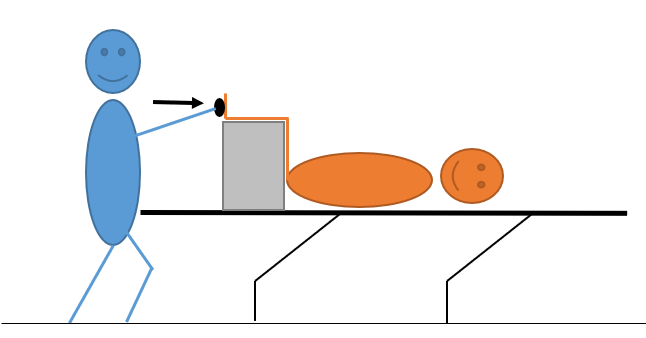 | | | | | Sitting at the stool at the side of the feet of the patient, back against the wall, face towards the patient. Elbows resting at the couch. Using two hands for the measurement.  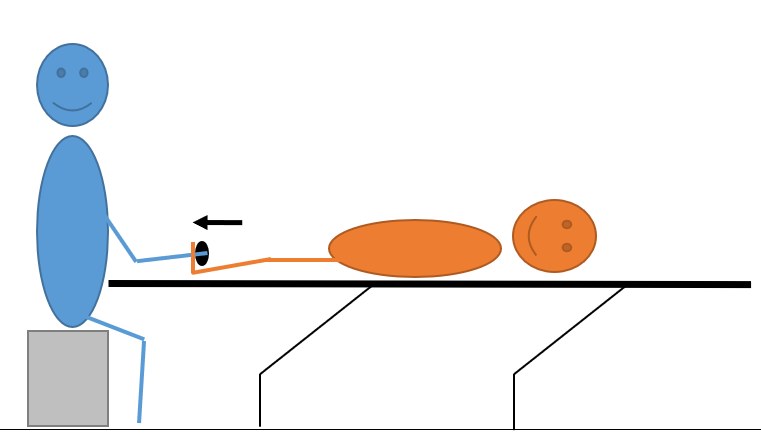 | |
| **Stabilization** | Hold the patient stabilized in the correct position manually at the hip and knee. | | | Hold the patient stabilized in the correct position manually at the hip and lower leg. | | | | | Hold the patient stabilized in the correct position manually at the hip and lower leg. | |
| **Position MicroFET** | Metatarsal heads, plantar side. | | | | | | | | Metatarsal heads, dorsal side. | |
| **Lever arm (m)** |  |  | | |  | |  | |  |  |
|  | *Left* | | *Right* | | | *Left* | | *Right* | *Left* | *Right* |
| **Power (N)** |  | |  | | |  | |  |  |  |
|  |  | |  | | |  | |  |  |  |
|  |  | |  | | |  | |  |  |  |
