# Supplementary material for: A randomized crossover study of functional electrical stimulation during walking in spastic cerebral palsy: the FES on participation (FESPa) trial
Source: BMC Pediatr. 2022 Jan 13;22:37. doi: 10.1186/s12887-021-03037-9 (PMC8756646; doi:10.1186/s12887-021-03037-9)
Supplement: Supplementary file 2 — Additional file 2. [file 12887_2021_3037_MOESM2_ESM.pdf]

Informatie voor deelname aan onderzoek

Functionele elektrostimulatie tijdens het lopen bij kinderen met cerebrale parese

Inleiding
Hoi,

Je krijgt deze brief omdat je cerebrale parese hebt en daardoor moeilijker loopt; met een klapvoet. Wij vragen jou of je mee wil doen aan een onderzoek. Er doen 25 kinderen mee van 4 tot 18 jaar. Je beslist samen met je ouders/verzorgers of je mee wilt doen. Als je niet wil meedoen, hoef je dat niet te doen. Je behandeling blijft dan hetzelfde.
Heb je na het lezen van de informatie nog vragen? Dan kan je terecht bij de onderzoekers (dokter Jeroen Vermeulen en dokter Irene Moll).

1. Wat is het doel van het onderzoek?
Het doel van het onderzoek is om de werking van een apparaat te bekijken en te vergelijken met spalken of aangepaste schoenen. Dit apparaat geeft tijdens het lopen kleine stroompjes zodat je je voet beter kan optillen. Dit heet ‘FES’ (functionele elektrostimulatie). Dit apparaat zit met een band net onder je knie. Hieronder staat een foto. We willen kijken of dit een goede behandeling voor jou is.

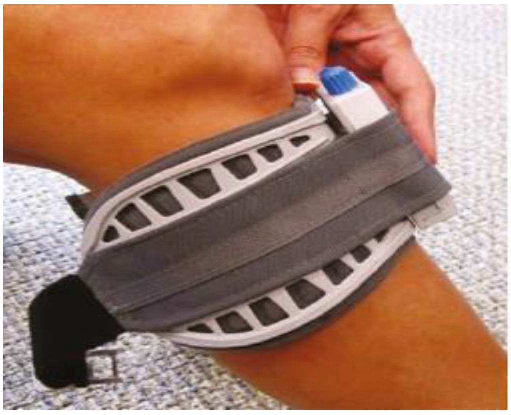
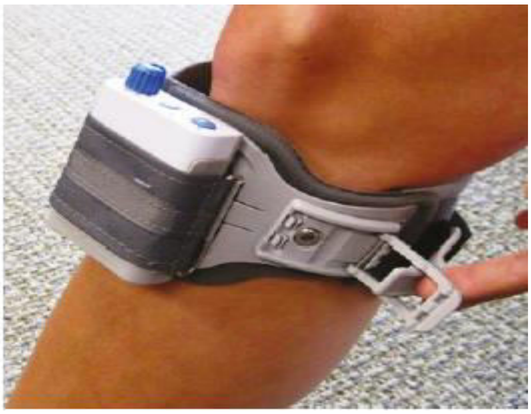

2. Waarom?
Bij cerebrale parese kunnen de hersenen de spieren niet helemaal goed aansturen en zijn niet alle spieren even sterk. FES kan misschien helpen bij lopen en je spieren sterker maken.

3. Hoe wordt het onderzoek uitgevoerd?

Eerst kijken we of je echt mee kan doen, door wat oefeningen met je benen te doen.
Als je meedoet, duurt dat 30 weken en zal je 5 keer naar het ziekenhuis moeten komen voor testen. Je krijgt een tijd (12 weken) behandeling met FES (nieuwe behandeling) waarbij je je spalk (als je die hebt) niet mag gebruiken én een tijd (18 weken) de standaardbehandeling die je nu ook al krijgt.
De testen bestaan uit opmeten, lopen en vragenlijsten invullen. In totaal duurt dit elke keer zo'n 3 tot 4 uur. Voor het looponderzoek plakken we bolletjes en plakkers op je huid. Hiervoor moet je ongeveer 20 minuten stilstaan. Daarna ga je met een harnas en eigen schoenen aan op een loopband lopen, met een groot TV scherm ervoor. Op de foto zie je hoe het er uit ziet.

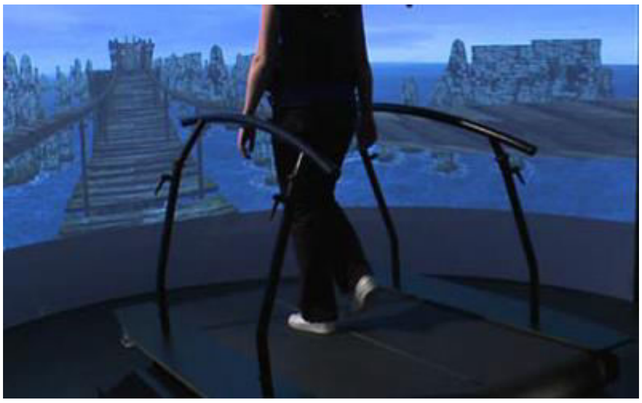

Soms zal het FES apparaat aan staan tijdens het lopen. Er wordt verder niets gedaan tijdens het lopen.
Daarnaast geven we je drie keer twee 'stappentellers' mee die je vervolgens 7 dagen op je been geplakt moet laten zitten.

4. Wat wordt er van jou verwacht?

De hele studie: je houdt je aan de behandeling. Dus een bepaalde tijd FES en een bepaalde tijd de behandeling die je nu al hebt.
Bij de metingen: sportkleding aandoen, meedoen met de oefeningen en stil staan als de bolletjes en plakkers worden geplakt. Vervolgens loop jij in de gymzaal en daarna op de loopband, met het harnas aan, en soms het FES apparaat aan. We zullen samen met je ouders vragenlijsten invullen. Je mag het apparaat niet kapot maken.

5. Wat kan je merken tijdens de studie?

Nadelen

Misschien krijg je een rode huid, kan je niet tegen de stroomstootjes of schaam je je voor het apparaat. Vertel het ons dan. Misschien val je iets vaker als je je spalk niet mag gebruiken.

Voordelen

Misschien kan je door FES je voet beter optillen en val je minder.

6. Als je niet (meer) wilt meedoen met het onderzoek

Als je niet meer mee wilt doen, moet je dat vertellen aan de onderzoekers. We zullen het onderzoek meteen stoppen bij jou. Dat geeft niets en je hoeft niet te vertellen waarom.

7. Wat gebeurt er als het onderzoek afgelopen is?

Na een meting worden alle bolletjes en plakkers weggehaald en mag je je gewone kleren weer aan. Bij de allerlaatste meting krijg je een klein cadeautje. Daarna mag je naar huis gaan.
Als alle 25 kinderen klaar zijn met het onderzoek, zullen we vertellen wat we hebben gevonden in alle metingen.

8. Heb je vragen?

De hoofdonderzoekers zijn dokter Jeroen Vermeulen (kinder neuroloog) en Irene Moll (basisarts) in het ziekenhuis Maastricht. Je ouders kunnen altijd contact opnemen als je vragen hebt, via telefoonnummer +31(0)43-387 5058. Voor onafhankelijk advies over meedoen aan dit onderzoek kun je terecht bij de onafhankelijke arts, mw. Drs. S.M. Koudijs, kinderneuroloog in het Erasmus ziekenhuis in Rotterdam, telefoonnummer +31(0)10 704 07 04. Je kunt altijd vragen stellen voor of tijdens het onderzoek.

Proefpersoneninformatie voor deelname aan medisch-wetenschappelijk onderzoek

Functionele elektrostimulatie tijdens het lopen bij kinderen met cerebrale parese

Officiële titel: Functionele elektrostimulatie van de enkel dorsaalflexoren tijdens het lopen bij kinderen met spastische cerebrale parese: een gerandomiseerde crossover interventie studie

Inleiding

Geachte jongen/meisje,

Je krijgt deze brief omdat je cerebrale parese hebt en daardoor moeilijker loopt; met een klapvoet. Wij vragen jou of je mee wil doen aan een onderzoek. Er doen 25 kinderen mee van 4-18 jaar, allemaal met cerebrale parese. Je beslist samen met je ouders/verzorgers of je mee wilt doen. Als je niet wil meedoen, hoef je dat niet te doen. Dat heeft geen gevolgen.
In deze brief staat uitleg over het onderzoek, zodat je goed kan beslissen. Heb je na het lezen van de informatie nog vragen? Dan kan je terecht bij de onderzoekers (dokter Jeroen Vermeulen en dokter Irene Moll).

1. Wat is het doel van het onderzoek?

Het doel van het onderzoek is om de werking van een apparaat te onderzoeken en te vergelijken met spalken (orthesen of beugels) of aangepaste schoenen. Dit apparaat geeft tijdens het lopen kleine stroompjes zodat je je voet beter kan optillen. Dit heet 'FES': functionele elektrostimulatie. Dit apparaat zit met een band net onder je knie vast en lijkt qua grootte op een klein, dik mobieltje. Hieronder staat een foto. We willen kijken of je hierdoor beter en misschien verder kan lopen, en daardoor aan meer dingen kan meedoen. Nu betaalt de verzekering dit apparaat niet, maar wel aangepaste schoenen of spalken als je die nodig hebt. Misschien is FES voor jou beter-dan een spalk of aangepaste schoen.

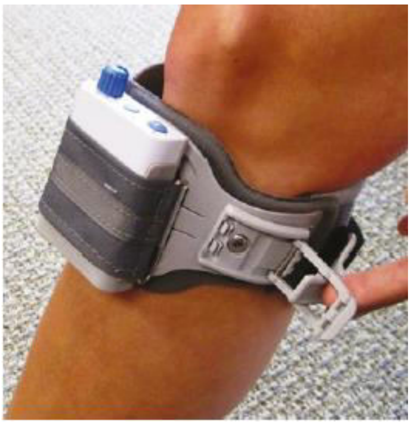

2. Waarom?

Bij cerebrale parese kunnen de hersenen de spieren niet helemaal goed aansturen en zijn niet alle spieren even sterk. Hierdoor lopen kinderen met cerebrale parese anders dan gezonde kinderen. Een behandeling hiervoor is een spalk of speciale schoen, waardoor je voet bijvoorbeeld in de goede houding wordt gezet. Door deze spalk of speciale schoen kan je voet minder bewegen. Bij FES kun je je voet normaal bewegen en worden je spieren misschien sterker. .

3. Hoe wordt het onderzoek uitgevoerd?

Eerst kijken we of je echt mee kan doen, door wat oefeningen met je benen. Soms gebeurt het dat we bij testen iets vinden waar een (andere) dokter nog verder naar moet kijken. Dan zullen we je dat vertellen.

Als je meedoet, duurt dat 30 weken en zal je 5 keer naar het ziekenhuis moeten komen voor testen. Je krijgt 12 weken lang behandeling met FES (nieuwe behandeling) waarbij je je spalk (als je die hebt) niet mag gebruiken, en 18 weken de standaardbehandeling die je nu ook al krijgt. Voor de volgorde van de behandeling wordt geloot.

De testen bestaan uit opmeten, lopen en vragenlijsten invullen. In totaal duurt dit elke keer zo'n 3 tot 4 uur, behalve het eerste kortere bezoek. Voor het looponderzoek plakken we bolletjes en plakkers op de huid. Hiervoor moet je ongeveer 20 minuten stilstaan. De bolletjes worden herkend door de camera's die jouw looppatroon filmen. De plakkers meten de spieractiviteit. Op de loopband krijg je een harnas (een soort pakje van touw) aan om te zorgen dat je niet kan vallen. Je loopt op je eigen schoenen. Tijdens het lopen kijk je naar een groot TV scherm. Soms zal het FES apparaat aan staan tijdens het lopen. Er wordt verder niets gedaan tijdens het lopen. Het doet dus ook geen pijn. Sommige kinderen vinden het wel vervelend als de bolletjes en plakkers weer weggehaald worden.

Daarnaast geven we je drie keer twee 'stappentellers' mee die je vervolgens 7 dagen op je been geplakt moet laten zitten.

Hieronder staat een plaatje van het verloop van de studie en van de loopband met scherm.

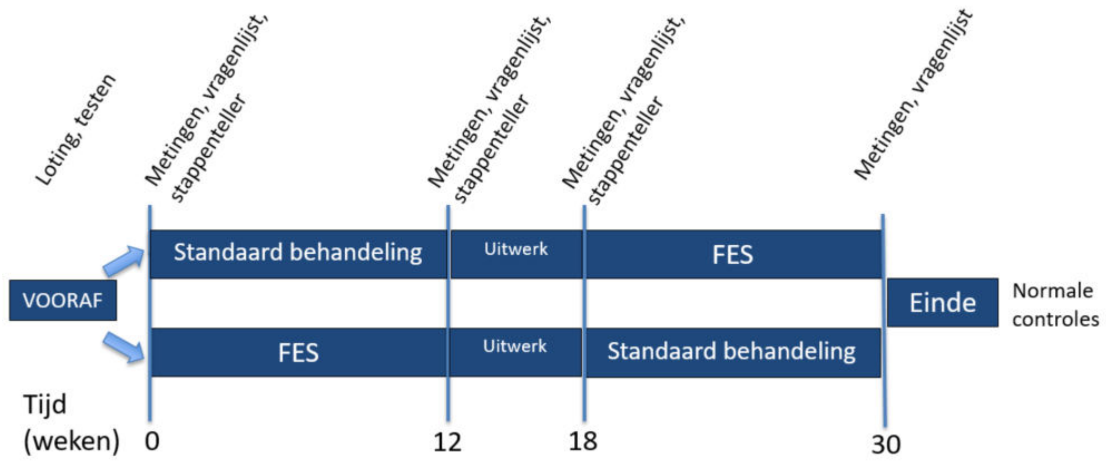

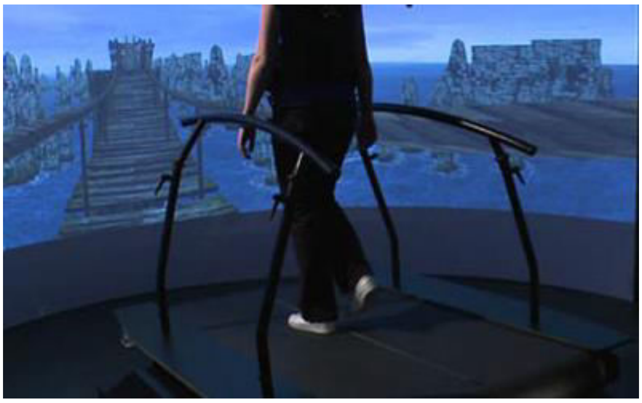

4. Wat wordt er van jou verwacht?

De hele studie: we verwachten dat jij de behandeling volgt die we voor een bepaalde tijd van je vragen. Dus een bepaalde tijd FES en een bepaalde tijd de behandeling die je nu al hebt.
Bij de metingen: het beste kun je sportkleding meenemen. We doen eerst samen oefeningen om je lenigheid en kracht te testen. Daarna plakken we de bolletjes en plakkers op terwijl je stilstaat. Vervolgens loop jij in de gymzaal en daarna op de loopband, met het harnas aan, en soms het FES apparaat aan. Tot slot vragen we je vragenlijsten in te vullen.
Er zijn een aantal regels:

- Je gebruikt het apparaat zoals uitgelegd en maakt het niet kapot.
- Je doet niet tegelijk aan een ander onderzoek mee.
- Je komt naar je afspraken in het ziekenhuis.

Als er iets veranderd in je medicijnen, je gezondheid of je adres/telefoonnummer moeten we dat i.v.m. het onderzoek weten. Laat je ouder(s)/verzorger(s) dan contact opnemen met de onderzoekers.

5. Wat kan je merken tijdens de studie?

Nadelen

In de studie wordt FES onderzocht. FES kan ook nadelen hebben, zoals:

- Het krijgen van een rode, geïrriteerde huid op de plek van het apparaat bij je knie
- Niet tegen de stimulatie (stroomstootjes) kunnen
- Het FES apparaat niet accepteren, omdat je je bijvoorbeeld schaamt
- Het zou kunnen dat je iets vaker valt als je je spalk niet mag gebruiken

We weten nog niet bij hoeveel mensen deze vervelende effecten optreden. We willen het graag weten als je iets vervelends ervaart, ook als het iets anders is dan hier genoemd staat.
Als je mee doet aan het onderzoek, krijg je de handleiding van het FES apparaat mee.
De studie zelf kan ook nadelen hebben, zoals extra moe zijn door de metingen en misschien school missen door de afspraken. Dit proberen we te voorkomen.

We vergelijken FES met een spalk of aangepaste schoenen, die je misschien al hebt. Dit kan ook nadelen hebben zoals het krijgen van drukplekken.

Voordelen

FES kan ook voordelen hebben. Dankzij het FES apparaat kan je waarschijnlijk je voet beter optillen, krijg je mogelijk betere balans en bewegingscontrole en val je misschien minder. Dit is niet zeker. Als je het apparaat fijn vindt, kunnen we kijken of je het na de studie kan blijven gebruiken.

6. Als je niet (meer) wilt meedoen met het onderzoek

Als je niet (meer) mee wilt doen, moet je dat vertellen aan de onderzoekers. We zullen het onderzoek meteen stoppen bij jou. Dat geeft niets en je hoeft niet te vertellen waarom.
Als er nieuwe informatie over het onderzoek is die belangrijk voor jou is, laat de onderzoeker dit aan je weten. Je wordt dan gevraagd of je blijft meedoen.

7. Wat gebeurt er als het onderzoek afgelopen is?

Na een meting worden alle bolletjes en plakkers weggehaald en mag je je gewone kleren weer aan. Bij de allerlaatste meting krijg je een klein cadeautje. Daarna mag je naar huis gaan.
Het onderzoek is afgelopen als álle metingen gedaan zijn, als je zelf wilt stoppen of als anderen (dokters, regering) beslissen dat het onderzoek (voor jou) stopt. De resultaten van jouw metingen gebruiken we voor het onderzoek. Als je wil, kan gekeken worden of je het FES apparaat kan blijven gebruiken. Tot nu toe betaalt de verzekering dit niet standaard.
Als alle 25 kinderen klaar zijn met het onderzoek en de gegevens zijn verwerkt, zullen we vertellen wat we hebben gevonden (de belangrijkste resultaten).

8. Wat gebeurt er met de onderzoekgegevens?

Wij zijn verplicht om jouw onderzoeksgegevens 15 jaar te bewaren. We gebruiken deze gegevens wellicht ook voor nieuw onderzoek in de toekomst. Daarvoor geef jij toestemming wanneer je meedoet aan dit onderzoek. Ook kunnen we je in de toekomst opnieuw vragen mee te doen aan onderzoek. Alle gegevens van jou die tijdens het onderzoek worden geregistreerd worden onder een uniek nummer anoniem geregistreerd. De koppeling van jouw naam en dat nummer (sleutel van de code) zijn uitsluitend bekend bij de hoofdonderzoekers en mensen die controles moeten uitvoeren. Wanneer er gegevens uit dit onderzoek openbaar worden gemaakt is dit niet herleidbaar naar jou.

9. Wie wordt geïnformeerd over deelname aan het onderzoek?

Wij sturen je huisarts en behandelend specialist en apotheker altijd een brief/e-mail om te laten weten dat je meedoet aan het onderzoek. Dit is voor je eigen veiligheid. Als je dit niet goed vindt, kun je niet meedoen aan dit onderzoek. In geval van onduidelijkheid kunnen we contact opnemen met je (huis)arts, bijvoorbeeld over je medische geschiedenis of over medicijngebruik.

9. Zijn er extra kosten of een vergoeding voor meedoen?

Er zijn geen extra kosten verbonden aan meedoen. Reiskosten worden vergoed. Je krijgt geen geld voor het meedoen, maar aan het einde van de studie wel en klein cadeau als bedankje.

11. Welke medisch-ethische toetsingscommissie heeft dit onderzoek goedgekeurd?

De Medisch-etische toetsingscommissie van het ziekenhuis in Maastricht azM/UM heeft dit onderzoek goedgekeurd.

10. Heb je vragen?

De hoofdonderzoekers zijn dokter Jeroen Vermeulen (kinderneuroloog) en Irene Moll (basisarts) in het ziekenhuis Maastricht. Jij of je ouders kunnen altijd contact opnemen als je vragen hebt, via telefoonnummer +31(0)43-387 5058. Voor onafhankelijk advies over meedoen aan dit onderzoek kun je terecht bij de onafhankelijke arts, mw. Drs. S.M. Koudijs, kinderneuroloog in het Erasmus ziekenhuis in Rotterdam, telefoonnummer +31(0)10 704 07 04. Je kunt altijd vragen stellen voor of tijdens het onderzoek.

11. Ondertekening toestemmingsformulier

Wanneer je voldoende bedenktijd (7 dagen) hebt gehad, worden jij en je ouders gevraagd te beslissen over deelname aan dit onderzoek. Indien je mee wilt doen, vragen we je daarvoor op papier een handtekening te zetten. Daarmee geef je aan dat je de informatie hebt begrepen en instemt met deelname aan het onderzoek. Ben je jonger dan 16 jaar, dan moeten je ouders/verzorgers ook allebei een formulier tekenen.
Het handtekeningenblad wordt door de onderzoeker bewaard. Jij krijgt een kopie of een tweede exemplaar van deze toestemmingsverklaring.

Dank voor je aandacht.

16. Bijlagen bij deze informatie

A. Contactgegevens
B. Toestemmingsformulieren
C. Handleiding FES apparaat (WalkAide)
D. Brochure ‘Medisch-wetenschappelijk onderzoek. Algemene informatie voor de proefpersoon’ (versie maart 2017)

Bijlage A: contactgegevens MUMC+

Hoofdonderzoeker:
Prof. dr. R.J. Vermeulen, kinderneuroloog
Afdeling Neurologie, Maastricht Universitair Medisch Centrum
Postbox 5800 6202 AZ Maastricht
Locatie: Oxfordgebouw | niveau 3 | kamernr. 3.R1.024
E jeroen.vermeulen@mumc.nl **T** +31(0)43-387 5058|**T**+31(0)387 7054 **F** +31(0)43-3877055 |

Dagelijks onderzoeker, onderzoeksarts:
Drs. I. Moll, promovendus
Universiteit Maastricht/MUMC+
Locatie: G2.202, Universiteitssingel 50
E irene.moll@maastrichtuniversity.nl **T** 0611922127.

Onafhankelijk arts:
Drs. S.M. Koudijs, kinderneuroloog
Erasmuc MC, Postbus 2060, 3000 CB Rotterdam
Bezoekadres: Wytemaweg 80, 3015 CN Rotterdam
E s.koudijs@erasmusmc.nl |**T** +31(0)10 704 07 04

Bij klachten:
Klachtenfunctionaris Maastricht UMC+
Postbus 5800, 6202 AZ Maastricht
E klachten@mumc.nl **T** 043-387 42 04 bereikbaarheid: maandag t/m donderdag van 10.00 tot 12.00 uur.

Indien nodig is er 24 uur per dag, 7 dagen per week, een dienstdoende kinderneuroloog bereikbaar via de centrale van het MUMC+, telefoonnummer: 043 387 6543.

Bijlage B: toestemmingsformulier proefpersoon vanaf 12 jaar

Functionele elektrostimulatie tijdens het lopen bij kinderen met cerebrale parese

- Ik heb de informatiebrief gelezen. Ook kon ik vragen stellen. Mijn vragen zijn voldoende beantwoord. Ik had genoeg tijd om te beslissen of ik meedoe.
- Ik weet dat meedoen vrijwillig is. Ook weet ik dat ik op ieder moment kan beslissen om toch niet mee te doen of te stoppen met het onderzoek. Daarvoor hoef ik geen reden te geven.
- Ik geef toestemming voor het informeren van mijn huisarts en evt. specialist(en) die mij behandelt en apotheker dat ik meedoe aan dit onderzoek
- Ik geef toestemming voor het opvragen van informatie bij mijn huisarts en/of specialist(en) die mij behandelt over eventuele onduidelijkheden.
- Ik weet dat sommige mensen mijn gegevens kunnen inzien en dat mijn gegevens eventueel voor vergelijkbaar toekomstig onderzoek gebruikt kunnen worden. Die mensen staan vermeld in deze informatiebrief.
- Ik geef toestemming voor het verzamelen en gebruiken van mijn gegevens de manier en voor de doelen die in de informatiebrief staan
- Ik geef toestemming om mijn gegevens op de onderzoekslocatie nog 15 jaar na dit onderzoek te bewaren.

- Ik wil meedoen aan dit onderzoek.

Naam proefpersoon:
Handtekening: Datum : __ / __ / __

Ik verklaar dat ik deze proefpersoon volledig heb geïnformeerd over het genoemde onderzoek.

Als er tijdens het onderzoek informatie bekend wordt die de toestemming van de proefpersoon zou kunnen beïnvloeden, dan breng ik hem/haar daarvan tijdig op de hoogte.

Naam onderzoeker (of diens vertegenwoordiger):
Handtekening: Datum: __ / __ / __

Aanvullende informatie is gegeven door:
Naam:
Functie:

Handtekening: Datum: __ / __ / __

----

* Doorhalen wat niet van toepassing is.

De proefpersoon krijgt een volledige informatiebrief mee, samen met een kopie van het getekende toestemmingsformulier.

Bijlage: Toestemmingsformulier ouders of voogd bij kinderen t/m 15 jaar

Functionele elektrostimulatie tijdens het lopen bij kinderen met cerebrale parese

Ik ben gevraagd om toestemming te geven voor deelname van de volgende persoon/mijn kind aan dit medisch-wetenschappelijke onderzoek:

Naam proefpersoon (kind): Geboortedatum: __ / __ / __

- Ik heb de informatiebrief voor de ouders/verzorgers gelezen. Ook kon ik vragen stellen. Mijn vragen zijn voldoende beantwoord. Ik had genoeg tijd om te beslissen of ik wil dat mijn kind meedoet.
- Ik weet dat meedoen vrijwillig is. Ook weet ik dat ik op ieder moment kan beslissen dat mijn kind toch niet meedoet. Daarvoor hoef ik geen reden te geven.
- Ik geef toestemming voor het informeren van de huisarts/specialist(en) die mijn kind behandelt en diens apotheker dat mijn kind meedoet aan dit onderzoek.
- Ik geef toestemming voor het opvragen van informatie bij de huisarts/specialist(en) die mijn kind behandelt over eventuele onduidelijkheden.
- Ik weet dat sommige mensen de gegevens van mijn kind kunnen inzien en dat mijn gegevens eventueel voor vergelijkbaar toekomstig onderzoek gebruikt kunnen worden. Die mensen staan vermeld in deze informatiebrief.
- Ik geef toestemming voor gebruik van de gegevens op de manier en voor de doelen die in de informatiebrief staan.
- Ik geef toestemming om de gegevens van mijn kind op de onderzoekslocatie nog 15 jaar na dit onderzoek te bewaren.

- Ik ga ermee akkoord dat deze persoon/mijn kind meedoet aan dit onderzoek.

Naam ouder/voogd**:
Handtekening: Datum: __ / __ / __
Naam ouder/voogd**:
Handtekening: Datum: __ / __ / __

Ik verklaar hierbij dat ik bovengenoemde persoon/personen volledig heb geïnformeerd over het genoemde onderzoek.

Als er tijdens het onderzoek informatie bekend wordt die de toestemming van de ouder of voogd zou kunnen beïnvloeden, dan breng ik hem/haar daarvan tijdig op de hoogte.

Naam onderzoeker (of diens vertegenwoordiger):

Handtekening: Datum: __ / __ / __

Aanvullende informatie is gegeven door:

Naam:

Functie:

Handtekening Datum: __ / __ / __

----

* Doorhalen wat niet van toepassing is.

** Als het kind jonger dan 16 jaar is, ondertekenen de ouders die het gezag uitoefenen of de voogd dit formulier. Kinderen van 12 t/m 15 jaar die zelfstandig beslissingen kunnen nemen (wilsbekwaam zijn), moeten daarnaast zelf een formulier ondertekenen.

De ouder/voogd krijgt een volledige informatiebrief mee, samen met een kopie van het getekende toestemmingsformulier.

Proefpersoneninformatie voor deelname aan medisch-wetenschappelijk onderzoek

Functionele elektrostimulatie tijdens het lopen bij kinderen met cerebrale parese

Officiële titel: Functionele elektrostimulatie van de enkel dorsaalflexoren tijdens het lopen bij kinderen met spastische cerebrale parese: een gerandomiseerde crossover interventie studie

Inleiding

Geachte heer/mevrouw,

U ontvangt deze brief omdat uw kind cerebrale parese heeft en daarbij loopt met een klapvoet. Wij vragen u of uw kind mee wil doen aan een medisch-wetenschappelijk onderzoek. U beslist samen met uw kind of uw kind mee wilt doen. Meedoen is vrijwillig. Om mee te doen is wel uw schriftelijke toestemming nodig.
Voordat u beslist of uw kind wilt meedoen aan dit onderzoek, krijgt u uitleg over wat het onderzoek inhoudt. Lees deze informatie rustig door en vraag de onderzoeker uitleg als u vragen heeft. U kunt ook de onafhankelijk deskundige, die aan het eind van deze brief genoemd wordt, om aanvullende informatie vragen. U kunt er ook over praten met uw partner, vrienden of familie. Verdere informatie over meedoen aan zo'n onderzoek staat in de bijgevoegde brochure 'Medisch-wetenschappelijk onderzoek'.

1. Algemene informatie

Dit onderzoek wordt gedaan door de afdeling Kinderneurologie van het Maastricht Universitair Medisch Centrum (MUMC+), in samenwerking met artsen en therapeuten uit revalidatiecentra uit de buurt.
Voor dit onderzoek zijn 25 proefpersonen van 4-18 jaar met unilaterale spastische cerebrale parese nodig en deze personen zullen in Nederland worden gezocht.
De medisch-ethische toetsingscommissie azM/UM (Maastricht) heeft dit onderzoek goedgekeurd. Algemene informatie over de toetsing van onderzoek vindt u in de brochure 'Medisch-wetenschappelijk onderzoek'.

2. Doel van het onderzoek

Het doel van het onderzoek is om de effectiviteit van een medisch hulpmiddel, het FES apparaat, te onderzoeken en te vergelijken met de huidige standaard behandeling bij kinderen met spastische cerebrale parese zoals orthesen. Het FES apparaat is een goedgekeurd hulpmiddel en wordt gedragen net onder de knie. Het geeft tijdens het lopen elektrische stroompjes af om spieren te activeren zodat de voet beter opgetild wordt. Dit principe wordt 'functionele elektrostimulatie' (FES) genoemd. Hieronder ziet u een foto van het apparaat. Het FES apparaat

wordt nog niet door de Nederlandse zorgverzekeraars vergoed. De huidige standaardbehandeling bestaat uit fysiotherapie, aangepast schoeisel en/of orthesen – dit wordt wel vergoed. Dit onderzoek wil vooral bekijken of kinderen met unilaterale spastische cerebrale parese door FES beter hun persoonlijke doelen kunnen bereiken; of ze activiteiten (zoals lopen) met minder beperkingen kunnen uitvoeren en of ze dan ook meer kunnen participeren (deelnemen) aan het leven. Alle deelnemers zullen een periode de FES behandeling krijgen en een periode de standaardbehandeling.

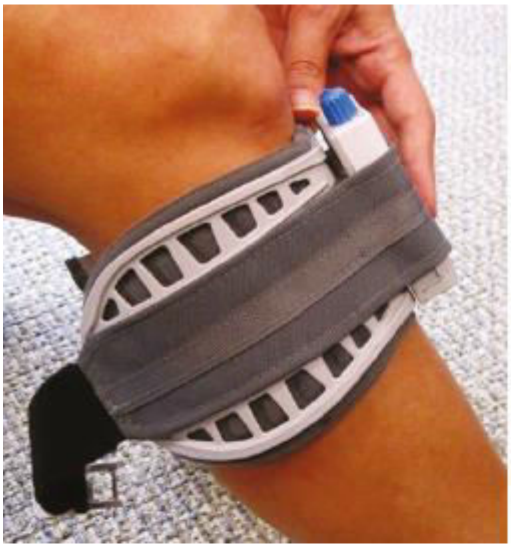

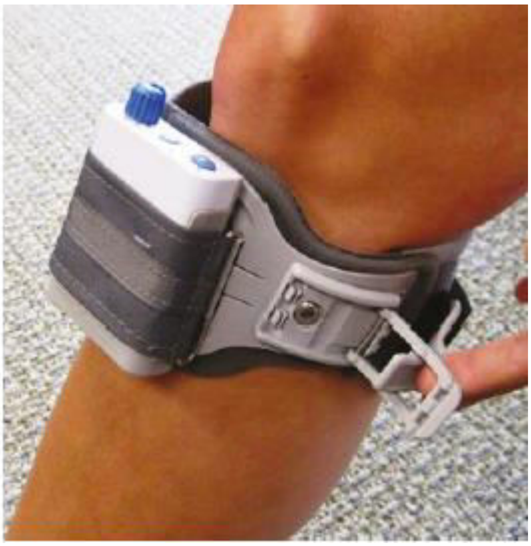

3. Achtergrond van het onderzoek

Cerebrale parese geeft vaak spasticiteit, waarbij de kuitspieren sterker zijn dan scheenspieren. Hierdoor kan patiënt de tenen en voet niet goed optillen en met een klapvoet lopen. Een behandeling hiervoor is een enkel-voet orthese (spalk), waardoor de juiste houding van de voet ondersteund wordt, of speciale schoenen. Bewegingen worden zo echter ook beperkt. FES is een andere aanpak, waarbij de zwakke spieren gestimuleerd worden, in plaats van de sterkere spieren beperkt. Uit onderzoek blijkt dat de voet hierdoor beter opgetild wordt en deze spierkracht toeneemt. De praktische betekenis voor patiënten is niet goed onderzocht.

4. Wat meedoen inhoudt

Als uw kind meedoet, duurt dat in totaal 30 weken voor uw kind, met meerdere ziekenhuisbezoeken. Er zijn 5 ziekenhuisbezoeken die elk 3 tot 4 uur duren, behalve het eerste kortere bezoek. Daarnaast zal het tijd kosten voor uw kind om te wennen aan de (nieuwe) FES behandeling en dit goed in te stellen met behulp van therapeuten.

Geschiktheidsonderzoek

Eerst bepalen we of uw kind kan meedoen. De onderzoeker doet lichamelijk onderzoek om dit te kunnen beslissen. Dit zijn testen die uw kind waarschijnlijk vaker meegemaakt heeft bij een arts.

Soms vinden we bij het geschiktheidsonderzoek iets dat verder medisch moet worden onderzocht. We vertellen u en uw kind dit altijd. Verder onderzoek gebeurt door de eigen huisarts of specialist. De kosten hiervan vallen onder uw eigen verzekering.

Behandeling met het FES apparaat

Uw kind gaat 12 weken lang het FES apparaat gebruiken, en 18 weken de standaard behandeling. Tijdens de FES periode kan een (eventuele) orthese niet gebruikt worden, omdat deze twee therapieën niet gelijktijdig kunnen worden toegepast. De helft van de proefpersonen krijgt eerst de FES behandeling, de andere helft eerst de standaard behandeling. Loting bepaalt welke behandeling uw kind eerst krijgt. Zie het stroomdiagram hieronder voor het verloop van de studie.

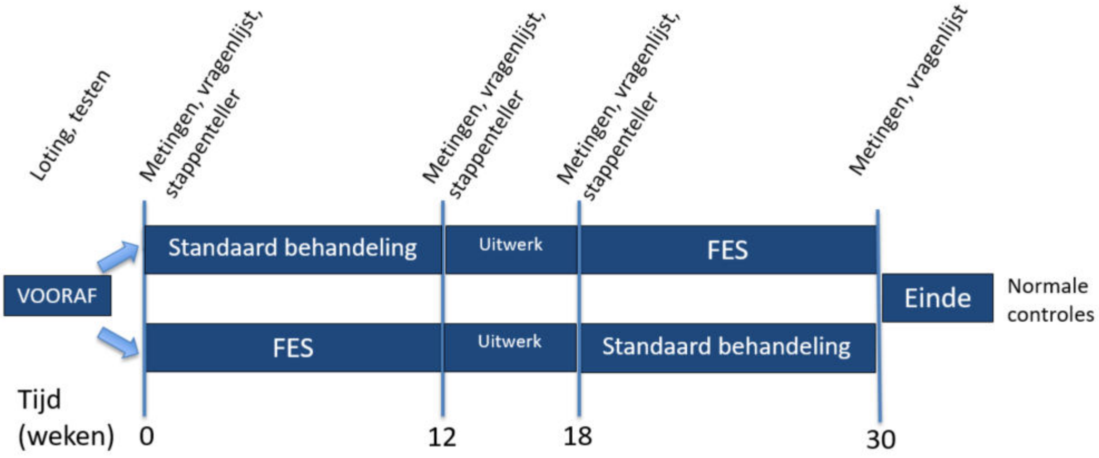

Bezoeken en metingen

Voor het onderzoek is het nodig dat uw kind 5 keer in 30 weken naar het ziekenhuis komt. Een bezoek duurt zo'n 3 tot 4 uur, behalve het eerste kortere bezoek. Er zal dan het volgende gebeuren:

- We doen een lichamelijk onderzoek - bij vier de bezoeken
- We doen een loop- & kracht analyse – bij vier bezoeken. Voor het looponderzoek plakken we bolletjes en plakkers op de huid. Hiervoor moet uw kind ongeveer 20 minuten stilstaan. De bolletjes worden herkend door de camera's die het looppatroon filmen. De plakkers meten de spieractiviteit. Uw kind zal vervolgens op een loopband lopen, met extra beveiliging van een harnas (dat vallen voorkomt) en de eigen schoenen om de voeten te beschermen. Tijdens het lopen kijkt uw kind naar een groot TV scherm. (Zie foto). Soms zal het FES apparaat aan staan tijdens het lopen. Na dit onderdeel worden de bolletjes en plakkers weer verwijderd, wat kinderen soms vervelend vinden.
- We laten vragenlijsten invullen over vallen, tevredenheid, mobiliteit en leven met beperkingen – bij vier bezoeken

- We geven u twee activiteitenmeters ('stappentellers') mee die uw kind vervolgens 7 dagen moet dragen – bij drie bezoeken

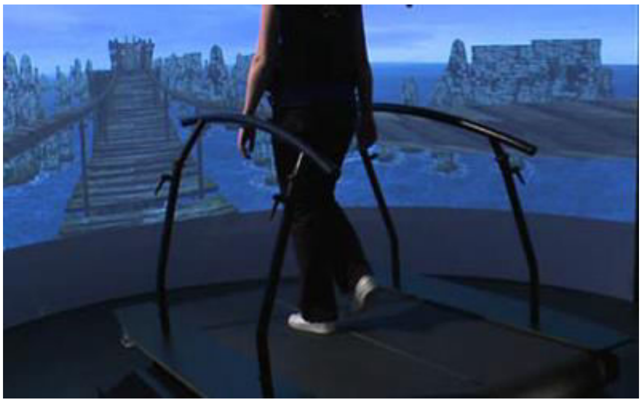

5. Wat wordt er van uw kind verwacht

Om het onderzoek goed te laten verlopen en voor de veiligheid van uw kind, is het belangrijk dat u zich aan de volgende afspraken houdt.

De afspraken zijn dat uw kind:

- Het hulpmiddel gebruikt volgens de uitleg.
- Niet ook nog aan een ander medisch-wetenschappelijk onderzoek meedoet.
- Afspraken voor bezoeken nakomt.

Het is belangrijk dat u contact opneemt met de onderzoeker:

- Voordat uw kind andere geneesmiddelen gaat gebruiken. Ook als dat homeopathische geneesmiddelen, natuurgeneesmiddelen, vitaminen en/of geneesmiddelen van de drogist zijn.
- Als uw kind in een ziekenhuis wordt opgenomen of behandeld.
- Als uw kind plotseling gezondheidsklachten krijgt.
- Als u(w kind) niet meer wilt meedoen aan het onderzoek.
- Als uw contactgegevens wijzigen.

Zwangerschap van een vrouwelijke deelnemer

Vrouwen die zwanger zijn of borstvoeding geven, kunnen niet meedoen aan dit onderzoek. Ook mogen vrouwen niet zwanger worden tijdens het onderzoek. Zwangerschap kan namelijk invloed hebben op de resultaten en het is niet precies onderzocht of er gevolgen zijn van FES voor het ongeboren kind. Wordt uw kind tijdens het onderzoek toch zwanger? Laat dit dan direct weten aan de onderzoeker.

6. Mogelijke ongemakken en complicaties

Het te onderzoeken hulpmiddel (FES apparaat) kan nadelige effecten hebben, namelijk:

- Huidirritatie
- Het niet kunnen verdragen van de elektrische stimulatie

- Problemen met het accepteren van het apparaat (schaamte, het apparaat te groot vinden)

Het is niet bekend bij hoeveel mensen deze nadelige effecten optreden. We vragen u het ons te melden als uw kind vervelende dingen ervaart omtrent de nieuwe behandeling, en direct contact op te nemen als de huid op de plek van de stimulatie beschadigd raakt. FES kan ook bijwerkingen hebben die nog onbekend zijn.
Doet uw kind mee aan het onderzoek? Dan krijgt u de handleiding van het FES apparaat mee.

Comparator
Ook de comparator (behandeling waarmee vergeleken wordt), in dit geval een enkel-voet orthese en/of aangepast schoeisel, kan nadelen hebben. De belangrijkste zijn het ontstaan van drukplekken. Omdat uw kind momenteel deze behandeling al krijgt, bent u hier wellicht al mee bekend.

Metingen
De metingen kunnen als vermoeiend worden ervaren, omdat uw kind zal moeten lopen (bijvoorbeeld zo ver mogelijk lopen in 6 minuten) en de maximale kracht zal moeten laten meten.

7. Mogelijke voor- en nadelen

Het is belangrijk dat u de mogelijke voor- en nadelen goed afweegt voordat jullie besluiten dat uw kind meedoet.

Voordelen van meedoen aan het onderzoek kunnen zijn:
Het te onderzoeken FES apparaat kan zorgen voor verbeterde voetheffing, balans en bewegingscontrole en wellicht minder vallen, maar zeker is dat niet. Indien uw kind voordeel ervaart van het apparaat, kan er een vergoeding bij de verzekeraar voor worden aangevraagd.

Nadelen van meedoen aan het onderzoek kunnen zijn:
- mogelijke huidirritatie (onder de knie)
- mogelijk vaker vallen in de FES periode, waarin de spalk niet gebruikt mag worden
- extra vermoeidheid door metingen in het ziekenhuis
- wellicht school missen door ziekenhuis afspraken – hier wordt zo veel mogelijk rekening mee gehouden
- het niet blij/tevreden zijn met het FES apparaat

Deelname aan het onderzoek betekent ook:
- dat u en uw kind extra tijd kwijt zijn;
- (extra) testen;
- dat uw kind afspraken heeft waaraan hij/zij zich moet houden;

Al deze zaken zijn hiervoor onder punt 4, 5 en 6 beschreven.

8. Verzet van uw kind (of degene die u vertegenwoordigt)

Het kan zijn dat uw kind/degene die u vertegenwoordigt zich tijdens het onderzoek verzet (niet meewerkt). De onderzoeker moet het onderzoek dan direct stoppen. Het is moeilijk om precies te omschrijven wat verzet is. Voor de start van het onderzoek wordt met u overlegd wat als verzet wordt gezien. De onderzoeker zal zich houden aan de Gedragscode verzet minderjarige.

9. Als uw kind niet wilt meedoen of wilt stoppen met het onderzoek

Jullie beslissen zelf of uw kind meedoet aan het onderzoek. Deelname is vrijwillig. Als uw kind niet wilt meedoen, wordt hij/zij op de gebruikelijke manier behandeld voor (problemen bij) cerebrale parese.
Als uw kind wel meedoet, kunt u zich altijd bedenken en toch stoppen, ook tijdens het onderzoek. Uw kind wordt dan weer op de gebruikelijke manier behandeld voor (problemen bij) cerebrale parese. U hoeft niet te zeggen waarom uw kind stopt. Wel moet u dit direct melden aan de onderzoeker.
De gegevens die tot dat moment zijn verzameld, worden gebruikt voor het onderzoek.
Als er nieuwe informatie over het onderzoek is die belangrijk voor u is, laat de onderzoeker dit aan u weten. U wordt dan gevraagd of u blijft meedoen.

10. Einde van het onderzoek

De deelname van uw kind aan het onderzoek stopt als

- alle bezoeken zoals beschreven onder punt 4 voorbij zijn
- u(w kind) zelf kiest om te stoppen
- uw kind zwanger wordt
- de onderzoeker het beter voor uw kind vindt om te stoppen
- MUMC+, de overheid of de beoordelende medisch-ethische toetsingscommissie, besluit om het onderzoek te stoppen.

Het hele onderzoek is afgelopen als alle deelnemers klaar zijn. Het FES apparaat dat tijdens de studie gebruikt wordt, is (ook na afloop van de studie) beschikbaar in Nederland maar wordt tot nu toe niet standaard vergoed door de zorgverzekering. De onderzoeker zal met u praten over de mogelijkheden voor verdere medische zorg.

Na het verwerken van alle gegevens informeert de onderzoeker u over de belangrijkste uitkomsten van het onderzoek.

11. Gebruik en bewaren van uw gegevens

Voor dit onderzoek is het nodig dat medische gegevens van uw kind worden verzameld en gebruikt. Elke proefpersoon krijgt een code die op de gegevens komt te staan. Naam en andere persoonlijke gegevens van uw kind die direct kunnen identificeren worden daarbij weggelaten.

Uw gegevens

Alle gegevens van uw kind blijven vertrouwelijk. Alleen de hoofdonderzoeker weet welke code uw kind heeft. Sommige mensen mogen uw medische en persoonlijke gegevens inzien. Dit is om te controleren of het onderzoek goed en betrouwbaar uitgevoerd is. Algemene informatie hierover vindt u in de brochure 'Medisch-wetenschappelijk onderzoek'.
Mensen die uw gegevens kunnen inzien zijn: het onderzoeksteam, de medisch-etische toetsingscomissie, de veiligheidscommissie die het onderzoek in de gaten houdt en de Inspectie voor de Gezondheidszorg. Zij houden de gegevens van uw kind geheim. Als u de toestemmingsverklaring ondertekent, geeft u toestemming voor het verzamelen, bewaren en inzien van medische en persoonlijke gegevens van uw kind.
De onderzoeker bewaart de gegevens van uw kind 15 jaar en mag deze gegevens gebruiken voor later extra onderzoek (over lopen bij cerebrale parese) indien van toepassing. Ook kunnen we uw kind in de toekomst opnieuw vragen aan onderzoek mee te doen.

Dit onderzoek staat ook in een overzicht van medisch-wetenschappelijke onderzoeken, namelijk https://clinicaltrials.gov/. Deze website bevat geen informatie die herleidbaar is tot u als persoon. Wel kan de website een samenvatting van de resultaten tonen. U vindt dit onderzoek onder 'Functional electrical stimulation during walking in cerebral palsy'.
Algemene informatie over de registratie van onderzoeken vindt u in de brochure 'Medisch-wetenschappelijk onderzoek'.

12. Verzekering voor proefpersonen

Voor iedereen die meedoet aan dit onderzoek is een verzekering afgesloten. De verzekering dekt schade door het onderzoek. Dit geldt voor schade die naar boven komt tijdens het onderzoek, of binnen vier jaar na het einde van het onderzoek. Niet alle schade is gedekt. In **bijlage B** vindt u meer informatie over de verzekering. Daar staat ook aan wie u schade kunt melden.

13. Informeren huisarts en behandelend specialist en apotheker

Wij sturen de huisarts van uw kind en behandelend specialist en apotheker altijd een brief/e-mail om te laten weten dat u w kind meedoet aan het onderzoek. Dit is voor de veiligheid van uw kind. Als u dit niet goed vindt, kunt u niet meedoen aan dit onderzoek. In geval van onduidelijkheid kunnen we contact opnemen met de (huis)arts, bijvoorbeeld over de medische geschiedenis of over medicijngebruik van uw kind.

14. Geen vergoeding voor meedoen

Het FES apparaat, extra testen en behandeling voor het onderzoek kosten u en uw kind niets. U(w kind) wordt niet betaald voor het meedoen aan dit onderzoek. Wel krijgt u een vergoeding voor de (extra) reiskosten en ontvangt uw kind na afloop van de studie een klein presentje als dank voor de deelname.

15. Heeft u vragen?

Bij vragen kunt u contact opnemen met het onderzoeksteam. Voor onafhankelijk advies over meedoen aan dit onderzoek kunt u terecht bij de onafhankelijke arts, mw. Drs. S.M. Koudijs. Zij weet veel over het onderzoek, maar heeft niets te maken met dit onderzoek.
Bij klachten kunt u het beste terecht bij de Klachtenopvang van het MUMC+. Alle gegevens vindt u in **bijlage A**: Contactgegevens.

16. Ondertekening toestemmingsformulier

Wanneer u voldoende bedenktijd (7 dagen) heeft gehad, worden u en uw kind gevraagd te beslissen over deelname aan dit onderzoek. Indien u toestemming geeft, zullen wij u vragen deze op de bijbehorende toestemmingsverklaring schriftelijk te bevestigen. Door uw schriftelijke toestemming geeft u aan dat u de informatie heeft begrepen en instemt met deelname aan het onderzoek. Vanaf de leeftijd van 12 jaar moeten kinderen zelf ook schriftelijk toestemming geven. Het handtekeningenblad wordt door de onderzoeker bewaard. U krijgt een kopie of een tweede exemplaar van deze toestemmingsverklaring.

Dank voor uw aandacht.

16. Bijlagen bij deze informatie

A. Contactgegevens
B. Informatie over de verzekering
C. Handleiding FES apparaat (WalkAide)
D. Toestemmingsformulieren
E. Brochure ‘Medisch-wetenschappelijk onderzoek. Algemene informatie voor de proefpersoon’ (versie maart 2017)

Bijlage A: contactgegevens MUMC+

Hoofdonderzoeker:
Prof. dr. R.J. Vermeulen, kinderneuroloog
Afdeling Neurologie, Maastricht Universitair Medisch Centrum
Postbox 5800 6202 AZ Maastricht
Locatie: Oxfordgebouw | niveau 3 | kamernr. 3.R1.024
E jeroen.vermeulen@mumc.nl **T** +31(0)43-387 5058|**T**+31(0)387 7054 **F** +31(0)43-3877055 |

Dagelijks onderzoeker, onderzoeksarts:
Drs. I. Moll, promovendus
Universiteit Maastricht/MUMC+
Locatie: G2.202, Universiteitssingel 50
E irene.moll@maastrichtuniversity.nl **T** 0611922127.

Onafhankelijk arts:
Drs. S.M. Koudijs, kinderneuroloog
Erasmuc MC, Postbus 2060, 3000 CB Rotterdam
Bezoekadres: Wytemaweg 80, 3015 CN Rotterdam
E s.koudijs@erasmusmc.nl |**T** +31(0)10 704 07 04

Bij klachten:
Klachtenfunctionaris Maastricht UMC+
Postbus 5800, 6202 AZ Maastricht
E klachten@mumc.nl **T** 043-387 42 04 bereikbaarheid: maandag t/m donderdag van 10.00 tot 12.00 uur.

Indien nodig is er 24 uur per dag, 7 dagen per week, een dienstdoende kinderneuroloog bereikbaar via de centrale van het MUMC+, telefoonnummer: 043 387 6543.

Bijlage B: informatie over de verzekering

INFORMATIE PROEFPERSONENVERZEKERING **azM is opdrachtgever**

MODEL 1 VERZEKERINGSVERKLARING
voor het hierna te noemen wetenschappelijk onderzoek

'Functional electrical stimulation of the ankle dorsiflexors during walking in children with unilateral spastic cerebral palsy: a randomized crossover intervention study'

Voor iedereen die meedoet aan dit onderzoek, heeft het academisch ziekenhuis Maastricht, hierna te noemen azM, een verzekering afgesloten. De verzekering dekt schade door deelname aan het onderzoek. Dit geldt voor schade tijdens het onderzoek of binnen vier jaar na het einde ervan. Schade moet u binnen die vier jaar aan de verzekeraar hebben gemeld.

De verzekering dekt niet alle schade. Onderaan deze tekst staat in het kort welke schade niet wordt gedekt.
Deze bepalingen staan in het Besluit verplichte verzekering bij medisch-wetenschappelijk onderzoek met mensen. Dit besluit staat op www.ccmo.nl, de website van de Centrale Commissie Mensgebonden Onderzoek (zie 'Bibliotheek' en dan 'Wet- en regelgeving').

Bij schade kunt u direct contact leggen met de verzekeraar:

De verzekeraar van het onderzoek is:<br>Naam: CNA Insurance Company Ltd.<br>Adres: World Trade Centre, Strawinskylaan 703, 1077 XX Amsterdam<br>Telefoonnummer: 020-5737272<br>Polisnummer: 10174880<br>Contactpersoon: Esther van Herk, Snr Claims Examiner<br>E-mail: Esther.VanHerk@cnaeurope.com<br>Telefoonnummer: 020-5737274

De verzekering biedt een dekking van ten minste € 650.000 per proefpersoon en ten minste € 5.000.000 voor het hele onderzoek (en ten minste € 7.500.000 per jaar voor alle onderzoeken van het MUMC+).

De verzekering dekt de volgende schade **niet**:
- schade door een risico waarover u in de schriftelijke informatie bent ingelicht. Dit geldt niet als het risico zich ernstiger voordoet dan was voorzien of als het risico heel onwaarschijnlijk was;
- schade aan uw gezondheid die ook zou zijn ontstaan als u niet aan het onderzoek had meegedaan;
- schade door het niet (volledig) opvolgen van aanwijzingen of instructies;
- schade aan uw nakomelingen, als gevolg van een negatief effect van het onderzoek op u of uw nakomelingen;
- schade door een bestaande behandelmethode bij onderzoek naar bestaande behandelmethoden.

Bijlage D: Toestemmingsformulier ouders of voogd bij kinderen t/m 15 jaar

Functionele elektrostimulatie tijdens het lopen bij kinderen met cerebrale parese

Ik ben gevraagd om toestemming te geven voor deelname van de volgende persoon/mijn kind aan dit medisch-wetenschappelijke onderzoek:

Naam proefpersoon (kind): Geboortedatum: __ / __ / __

- Ik heb de informatiebrief voor de ouders/verzorgers gelezen. Ook kon ik vragen stellen. Mijn vragen zijn voldoende beantwoord. Ik had genoeg tijd om te beslissen of ik wil dat mijn kind meedoet.
- Ik weet dat meedoen vrijwillig is. Ook weet ik dat ik op ieder moment kan beslissen dat mijn kind toch niet meedoet. Daarvoor hoef ik geen reden te geven.
- Ik geef toestemming voor het informeren van de huisarts/specialist(en) die mijn kind behandelt en diens apotheker dat mijn kind meedoet aan dit onderzoek.
- Ik geef toestemming voor het opvragen van informatie bij de huisarts/specialist(en) die mijn kind behandelt over eventuele onduidelijkheden.
- Ik weet dat sommige mensen de gegevens van mijn kind kunnen inzien en dat mijn gegevens eventueel voor vergelijkbaar toekomstig onderzoek gebruikt kunnen worden. Die mensen staan vermeld in deze informatiebrief.
- Ik geef toestemming voor gebruik van de gegevens op de manier en voor de doelen die in de informatiebrief staan.
- Ik geef toestemming om de gegevens van mijn kind op de onderzoekslocatie nog 15 jaar na dit onderzoek te bewaren.

- Ik ga ermee akkoord dat deze persoon/mijn kind meedoet aan dit onderzoek.

Naam ouder/voogd**:
Handtekening: Datum: __ / __ / __
Naam ouder/voogd**:
Handtekening: Datum: __ / __ / __

Ik verklaar hierbij dat ik bovengenoemde persoon/personen volledig heb geïnformeerd over het genoemde onderzoek.

Als er tijdens het onderzoek informatie bekend wordt die de toestemming van de ouder of voogd zou kunnen beïnvloeden, dan breng ik hem/haar daarvan tijdig op de hoogte.

Naam onderzoeker (of diens vertegenwoordiger):
Handtekening: Datum: __ / __ / __

Aanvullende informatie is gegeven door:
Naam:
Functie:
Handtekening Datum: __ / __ / __

----

* Doorhalen wat niet van toepassing is.
** Als het kind jonger dan 16 jaar is, ondertekenen de ouders die het gezag uitoefenen of de voogd dit formulier. Kinderen van 12 t/m 15 jaar die zelfstandig beslissingen kunnen nemen (wilsbekwaam zijn), moeten daarnaast zelf een formulier ondertekenen.

De ouder/voogd krijgt een volledige informatiebrief mee, samen met een kopie van het getekende toestemmingsformulier.
